# Family perspective on caring for children with chronic conditions in the pre- and trans-pandemic contexts of COVID-19

**DOI:** 10.1590/1980-220X-REEUSP-2025-0448en

**Published:** 2026-07-24

**Authors:** Nayara Luiza Henriques, Maísa Mara Lopes Macêdo Breves, Melissa Joice de Abreu Felizardo, Gabriel Lucas Santos Galdino, Samylla Alves de Almeida, Elysângela Dittz Duarte

**Affiliations:** 1 Universidade Federal de Minas Gerais, Escola de Enfermagem, Departamento de Enfermagem Materno-Infantil e Saúde Pública, Belo Horizonte, MG, Brazil.

**Keywords:** Chronic Disease, Child Care, Family, COVID-19, Nursing Care

## Abstract

**Objective:**

To understand families’ perspectives on caring for children with chronic conditions in the pre- and trans-pandemic contexts of COVID-19 in light of the Family Management Style Framework.

**Methods:**

Longitudinal qualitative study conducted with 24 family caregivers of children aged 2 to 4 years. Data were collected through a socioeconomic questionnaire and semi-structured interviews in two stages: pre-pandemic (October 2019 to May 2020) and trans-pandemic (June 2020 to January 2021). Data were subjected to thematic analysis guided by the theoretical framework.

**Results:**

The family perspective guided care management over time and was modified by the pandemic. In the pre-pandemic context, it was centered on children’s strengths, directing actions toward developmental promotion and health maintenance. In the trans-pandemic context, the perspective focused on children’s frailties and vulnerabilities associated with the perceived threat of COVID-19, resulting in intensified surveillance and hygiene care.

**Conclusion:**

The family perspective guides care management and is sensitive to contextual changes, providing support for the qualification of professional practice in health crisis contexts.

## INTRODUCTION

The incorporation of new health technologies has contributed to the increasing specialization of maternal and child care^(1)^, resulting in reduced infant mortality rates and an increase in the number of children living with chronic conditions^(2)^. Chronic conditions in childhood are those of congenital or acquired origin, with biological, psychological, or cognitive bases, resulting in permanent or temporary functional limitations^(3)^.

In the home setting, families play a leading role in caring for children with chronic conditions (CCC)^(4)^. They face the constant challenge of adapting essential healthcare activities to their routines in order to maintain children’s health, including everything from feeding and hygiene to handling complex technological devices^(5)^. In addition to household tasks, caregivers frequently need to mobilize resources to regularly attend specialized appointments and reorganize family life during hospitalizations^(6)^. This set of responsibilities, inherent to these children’s life course, creates a daily routine characterized by overload and family stress^(7)^.

The magnitude of this issue is further intensified by the context in which the family is inserted. According to the Family Management Style Framework (FMSF)^(8)^, a theoretical model that supports the understanding of family management of chronic conditions in childhood, context constitutes a central dimension of family care, capable of modifying family living conditions and the way families perceive and manage care^(8)^.

An abrupt contextual change was experienced globally with the emergence of the Coronavirus Disease 2019 (COVID-19) pandemic in March 2020^(9)^. Due to the virus’s high transmission potential, governmental decrees established social distancing measures that resulted in the suspension of elective healthcare services and the closure of institutions such as schools and daycare centers, in addition to the adoption of protective measures such as mask use and intensified hand hygiene^(9)^. The implications of this contextual change affected everyone’s lives, especially those of families of CCC. In addition to performing the routine care required by the children, families incorporated new hygiene measures and experienced restricted social interaction, as well as suspension of healthcare follow-up and school activities. Thus, the health crisis context imposed additional demands on families that were already overloaded with tasks while simultaneously reducing their support networks^(10)^.

Given the substantial changes in the care of CCC promoted by the COVID-19 pandemic, especially when compared to the period prior to the pandemic, it is assumed that families modified their perspectives regarding children and their health condition, which directly impacted family care management. This is because care actions directed toward CCC, which represent the family’s active responses to children’s needs, depend on how these needs are recognized and perceived by families^(8)^. Thus, the family perspective regarding CCC’s capacities and needs directly shapes the way care is provided^(8)^.

An increasing number of studies have focused on the burden and emotional impact generated by caring for CCC. However, the available literature has predominantly addressed these aspects, placing less emphasis on analyzing families’ perspectives on care in distinct contexts marked by abrupt changes, such as the COVID-19 pandemic. Furthermore, although studies conducted during the pandemic addressed transformations in family care, they were mostly based on data collected after the establishment of a health crisis, limiting the analysis of family perspectives over time and in the face of contextual change, a gap addressed by this investigation.

Given this scenario, the following research question was defined: what are families’ perspectives on caring for a CCC in the pre- and trans-pandemic contexts of COVID-19? To answer this question, the study aimed to understand families’ perspectives on caring for CCC in the pre- and trans-pandemic contexts of COVID-19 in light of FMSF.

## METHOD

### Study Design

This is a longitudinal qualitative study^(11)^, developed in accordance with the transparency recommendations described in the Brazilian version of the Consolidated Criteria for Reporting Qualitative Research consensus tool^(12)^.

### Theoretical-Methodological Framework

For this investigation, FMSF^(8)^ was adopted as the theoretical framework, a model developed by nurses Kathleen Knafl and Janet Deatrick to understand how families perceive and actively respond to the care demands of a CCC considering the context in which they are inserted. Thematic analysis proposed by Braun and Clarke^(13)^ was adopted as the methodological framework, enabling the interpretation of research data based on previously defined categories grounded in the theoretical framework. In this study, the analysis focused on one of FMSF components, called “definition of the situation”, which allows apprehension of the family perspective regarding care for CCC.

### Participants

Twenty-four family caregivers of CCC aged between 2 and 4 years participated in the study. The children had been discharged from two Neonatal Intensive Care Units (NICUs) linked to a philanthropic hospital and a federal hospital, both located in a capital city in southeastern Brazil. The institutions were selected because they are national references within the Unified Health System for maternal and child care.

### Sample Definition and Selection Criteria

The sample was intentional and non-probabilistic, based on eligibility criteria. Family caregivers responsible for caring for CCC aged between 2 and 4 years were included, considering that neuropsychomotor developmental changes become more evident in this age group^(14)^. Caregivers had to be 18 years old or older, have feasible telephone contact, and reside in the same household as children, since they were considered key informants regarding the family dynamics involved in ensuring care for CCC in home environments; therefore, it was essential that they shared the same living space^(15)^. Caregivers with communication impairments or psychological and/or psychiatric conditions that made data collection unfeasible were excluded, as well as those who did not respond after three consecutive telephone contact attempts.

### Data Collection

The study was conducted in two stages corresponding to the two contexts investigated: stage 1 (pre-pandemic COVID-19 context), which covered the period from October 2019 to May 2020, during which the first phase of data production with the families took place, preceding the declaration of COVID-19 as a pandemic by the World Health Organization on March 11, 2020^(9)^; and stage 2 (during the pandemic or trans-pandemic context), referring to the period from June 2020 to January 2021, considered in this study as the interval during which data production occurred in the pandemic context, characterizing the course of this unprecedented global situation^(16)^.

Participant identification occurred during the first stage of the study (pre-pandemic context). To this end, all medical records of children discharged from the NICU between December 2016 and December 2017 were analyzed in order to include children aged between 2 and 4 years.

Based on the medical record analysis, 1,115 caregivers were identified, 852 from the philanthropic hospital and 263 from the federal hospital. Between October 2019 and May 2020, all caregivers were contacted by telephone to complete the Questionnaire for Identifying Children with Chronic Conditions – Revised (QuICCC-R), Brazilian version, an instrument used to screen for CCC^(17)^. Of the total caregivers identified, contact could not be established with 829 due to nonexistent or outdated telephone numbers. Thus, 286 caregivers were contacted. Of these, ten refused to participate; five reported that their children had died; and 218 had children who did not present chronic conditions according to the QuICCC-R, resulting in 53 caregivers participating in the first-stage data collection (pre-pandemic).

With the emergence of the COVID-19 pandemic, the study was expanded, with approval from the Ethics Committee, to include a second stage referring to the trans-pandemic context. Attempts were made to contact the same 53 caregivers who had participated in the first stage. Of these, nine no longer had the same telephone number; 13 did not respond after three contact attempts; and four declined participation. Thus, 27 caregivers remained, three of whom participated in a pilot study for validation of the interview guide and were not included in the data analysis. Therefore, the population defined for this study consisted of participants who were present in both stages, totaling 24 caregivers. It should also be emphasized that, at both moments of data collection, the same family member was considered the respondent.

In the first stage of the study, after confirmation of a child’s chronic condition, families were invited to participate in the study and interviews were scheduled. Initially, interviews were conducted in the families’ homes. However, due to the researchers’ difficulties traveling to the countryside of Minas Gerais, where most families lived, and with Ethics Committee approval, interviews began to be conducted by telephone. In the second stage, referring to the trans-pandemic context, considering the safety regulations implemented during the pandemic context, all interviews were conducted by telephone and audio-recorded after verbal consent from participants.

In both stages, a socioeconomic questionnaire was applied to collect information about children (sex, gestational and chronological age, and diagnosis) and the family member (relationship to children, age, ethnicity, educational level, marital status, occupation, and income). Subsequently, a semi-structured interview guide developed based on FMSF^(8)^ was used. In both phases, the focus of the interviews was the family perspective regarding care for CCC. Interviews were conducted by two researchers, both nurses, holding master’s degrees and pursuing doctoral studies in nursing, experienced in qualitative interviewing and with no prior relationship with families.

### Data Analysis and Treatment

The interviews were transcribed in full and subjected to deductive thematic analysis guided by the theoretical framework of FMSF. For this purpose, the analytical process proposed by Braun and Clarke^(13)^ was adopted, consisting of six phases: 1) Familiarization with the data through transcription and reading of the interviews; 2) Initial generation of preliminary codes based on the theoretical framework, encompassing the “definition of the situation” component of FMSF and its respective conceptual dimensions: child identity; illness view; management mindset; and parental mutuality; 3) Development of summaries of coded interview excerpts and recognition of patterns; 4) Review of identified patterns to ensure their distinction and consistency; 5) Elaboration of definitions for each identified pattern/theme; and 6) Description and analysis of results, relating them to the theoretical framework and the study objective.

### Ethical Aspects

The study was conducted in accordance with Resolutions 466/2012 and 580/2018 of the Brazilian National Health Council^(18,19)^, and was approved by the Research Ethics Committee under Opinion 3,508,414/2019 and Amendment 4,331,516/2020. Participants signed the Informed Consent Form in duplicate. To ensure confidentiality and anonymity, participants’ names were replaced in the interviews by alphanumeric codes using the letters M (“mother”), F (“father”), A (“aunt”), and C (“child”). The letters were followed by the number corresponding to the interview order (e.g., M4, F3, A1).

## RESULTS

### Characterization of Families and Children with Chronic Conditions

Most participating caregivers were mothers (n = 22). Only one interview was conducted with the father and another with the aunt of CCC. The respondents’ mean age was 34 years, ranging from 19 to 54 years. Most participants reported being married (n = 12), mixed-race (n=15), having completed higher education (n = 8), and living with their partners (n = 20) in the countryside of Minas Gerais (n = 16). Reported family income ranged between one and two minimum wages (n = 11), which at that time corresponded to R$1,100.00. Seven caregivers reported income below one minimum wage. Among the participating mothers, 14 were not employed and dedicated themselves to caring for CCC. In the trans-pandemic context, 17 participants reported having received some type of financial assistance. In terms of CCC, most were male (n = 15), with a mean age of 2 years and 8 months. Mean gestational age at birth was 31.8 weeks. The predominant medical diagnoses were neurological conditions (n = 10).

### Families’ Perspectives on Caring for Children with Chronic Conditions

Data analysis was directed toward understanding families’ perspectives on caring for CCC. For this purpose, the conceptual component “definition of the situation” from FMSF was used, as it enables understanding of how caregivers perceive children and their chronic condition, as well as how this perception influences care management. This component encompasses four dimensions explored in this study: child identity, illness view, management mindset, and parental mutuality.

**Figure 1:**
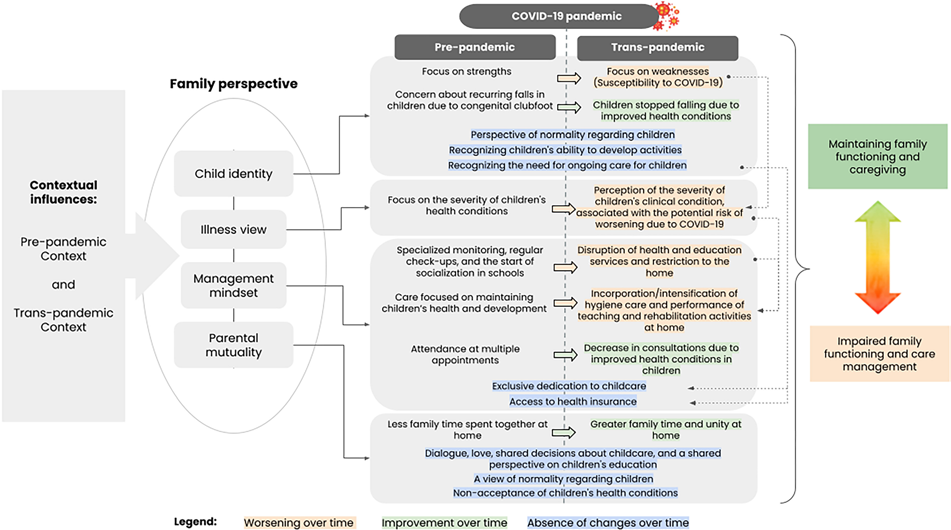
Family perspective on caring for children with chronic conditions in the pre- and trans-pandemic contexts of COVID-19 – Belo Horizonte, MG, Brazil, 2025.

A comparison was made between the information produced in the pre- and trans-pandemic contexts of COVID-19 in order to identify patterns and variations in families’ perspectives over time, highlighting situations of improvement, worsening, or absence of changes. This synthesis is represented in Figure 1, in which each dimension of the “definition of the situation” component is highlighted, while the dotted lines indicate the interconnection among them according to participants’ discourse. Following the figure, each dimension is presented in detail.

### Child Identity

According to FMSF, child identity refers to the family’s perspective regarding CCC, considering the recognition of children’s strengths and vulnerabilities^(8)^. In this study, in the pre-pandemic context, most families emphasized the children’s strengths, such as acquired abilities (standing, speaking, running, feeding themselves) and positive characteristics (independence, intelligence, good memory, and ease of learning). In the trans-pandemic context, however, this perspective changed and focused on vulnerabilities, particularly related to low immunity, pulmonary impairment, or recognition of CCC as susceptible to illness caused by COVID-19. Thus, worsening patterns in perspective were the most frequent in the face of contextual change.

The absence of changes in family perspective was evidenced by recognition that the children required continuous care and constant surveillance in both contexts studied. In other words, the focus remained on children’s vulnerabilities before and during the COVID-19 pandemic. This perception was more frequent among families of children with health conditions requiring more complex care. On the other hand, among those whose children presented less complex care demands, the perspective remained centered on children’s normality throughout both investigated moments.

Only one situation of improvement was identified, reported by M1, whose child presented difficulties walking due to congenital clubfoot in the pre-pandemic context. With resolution of the problem over time, there was a positive change in the family’s perception related to improvement in children’s health condition rather than to the pandemic context. Chart 1 presents information regarding families’ perspectives on CCC. The first column contains family perspectives along with the identification of families that reported them; the second presents the observed variation pattern (improvement, worsening, or absence of changes); and the third provides examples of statements illustrating these perspectives over time.

**Chart 1:** Child identity – Belo Horizonte, MG, Brazil, 2025.

Child identity			
Family perspective	Pattern of perspective variation	Empirical data	
		Pre-pandemic context	Trans-pandemic context
Children’s susceptibility to illness in the trans-pandemic context^(M1, M2, M3, M5, M6, M8, M9, M14, M16, M17, M18, M21 and M22)^	Worsening	*He’s a calm boy, not one of those kids who lives in the hospital. Health-wise, he’s calm [...] and he can already talk and stand up! (M1)*	*I’m really afraid he’ll catch the coronavirus. Because he has bronchitis, I think it’s easier for him to catch it. His immunity is lower, right? He’s in the high-risk group. (M1)*
The need for continued care in both contexts^(M8, M12 and M13)^	No modifications	*C12 requires 24-hour attention. For everything she does, she depends on someone. (M12)*	*She is a child who needs care all the time, for everything: to eat, to bathe, to move around. Her motor skills are completely compromised. (M12)*
A view of normality in both contexts^(M3, F4, M6, M7, A11, F15, M20, M23, M24)^	No modifications	*We already see C15 as a normal child because she is super intelligent. Her development is good. (F15)*	*Her development is excellent. During the pandemic, thank God, she is developing well. Everything is normal. (F15)*
Capacity for developing activities^(M2, M3, M4, M5, M6, M7, M9, M10, A11, M12, M13 and M14)^	No modifications	*Thank God, she has improved a lot. Nowadays, she can stand on her own; she takes a few steps; we see her progress. (M6)*	*In terms of speech, I have already been able to see some results. She already says “mommy”, “daddy”, “here”, “oops”, some monosyllabic things. (M6)*
Capacity for developing activities^(M1)^	Improvement	*C1 falls all the time and I’m afraid he’ll fall because he keeps hitting his face on the ground and I’m afraid he’ll hit his head. (M1)*	*He doesn’t fall as much as before. His feet were a little more turned inward, then they fixed it and he stopped falling. (M1)*

### Illness View

According to FMSF, illness view refers to the family’s understanding of children’s health condition^(8)^. In this study, it was identified that, during the pre-pandemic period, families recognized the severity of CCC’ health conditions. With the emergence of the pandemic, they began to perceive SARS-CoV-2 infection as an additional risk, constituting a direct threat to their children’s lives and intensifying the perception of severity regarding their health status. Thus, only worsening situations in the illness view were identified, as exemplified in Chart 2.

**Chart 2:** Illness view – Belo Horizonte, MG, Brazil, 2025.

Illness view			
Family perspective	Pattern of perspective variation	Empirical data	
		Pre-pandemic context	Trans-pandemic context
Perception of the severity of children’s clinical condition, associated with the potential risk of worsening due to COVID-19^(M1, M2, M3, M5, M6, M8, M9, M14, M16, M17, M18, M21, M22 and M24)^	Worsening	*C24 has a serious congenital heart defect, right? So, we’re following up properly. (M24)*	*If he catches the virus, it could worsen his heart condition even more; I don’t know if he’ll survive. (M24)*

### Management Mindset

According to FMSF, management mindset comprises the family’s perspective regarding facilitators and/or difficulties in carrying out CCC care regimen^(8)^. When comparing families’ discourses in both investigated contexts, worsening patterns in their perspectives were the most frequent. These were characterized by difficulties mentioned in relation to the COVID-19 pandemic context, such as intensified hygiene care to contain the disease and restriction of the family to home environments, which led to emotional repercussions such as anxiety and stress. The interruption of rehabilitation therapies and educational activities during the pandemic resulted in families having to take on and perform these activities at home — situations that led to a more negative perspective regarding care management. M13 reported a difficulty that remained in both contexts of the study related to the multiple appointments attended by her child. In the pre-pandemic context, the mother’s challenge was organizing herself to attend the appointments. In the trans-pandemic context, the difficulty was related to the interruption of care services.

Concerning facilitators for care that remained unchanged over time, the following stood out: continuity of therapies and healthcare follow-up even during the pandemic, the possibility of exclusive dedication to the children, and the presence of private health insurance. Only one situation of improvement was reported by M1, associated with the reduction in the number of appointments resulting from children’s clinical improvement, which facilitated care management. In this case, the improvement reflected children’s health condition rather than the pandemic context. Chart 3 exemplifies the information presented.

**Chart 3:** Management mindset – Belo Horizonte, MG, Brazil, 2025.

Management mindset			
Family perspective	Pattern of perspective variation	Empirical data	
		COVID-19 trans-pandemic context	
Difficulty regarding the interruption of health and education services^(M2, M5, M6, M7, M10, A11, M16 and M22)^	Worsening	*It’s been like this: a rush, right? More stressful, because there’s no school, right? So, they stay home more anxious. (M7)* *We had to stop the swimming lessons. The hospital’s outpatient clinic didn’t have appointments. Here at APAE, the doctor also stopped, so, well, the care became difficult. (M22)*	
Difficulty due to maintaining rehabilitation and education activities at home^(M2, M5, M6, M7, M10, A11, M16 and M22)^	Worsening	*There aren’t in-person appointments happening. So, in this case, there’s online physiotherapy, right? They call, they do video calls, and in this case, I’m the one who has to do physiotherapy. (M10)* *The school activities come to me to teach. It’s not easy, you know? (C6)*	
Difficulty due to increased hygiene measures^(M1, M3, M8, M9, M23 and M24)^	Worsening	*I’m being more careful with C1. I don’t go out as much as before, I’m always washing his little hands; when I go out, I get home and change his clothes right away, I give him a bath. My care for him has doubled. (M1)* *I wipe everything down every day with bleach to disinfect. I spray alcohol on C8’s things. Now, during the pandemic, it’s very difficult to maintain her care and these precautions, but I’m maintaining them all. (M8)*	
Difficulty due to children and family being confined to the home^(M1, M3, M7, M10, M13, M14, M17 and M19)^	Worsening	*Being stuck at home more is bad, isn’t it? We end up getting more stressed. We can’t go out much, we can’t have freedom. (M1)* *Ah, there was a change, right? Because we all stayed inside the house, especially for me. For me, it’s very tiring and stressful. (M13)*	
Ease of maintaining therapies during the pandemic^(M1, M2, M3, M6. M7, M9, A11)^	No modifications	*Thank God, equine therapy is working. It’s the only activity allowed in my city. (M3)* *They receive follow-up care at APAE, and it’s been very important for them now during the pandemic. (M7)*	
Ease of access for children through the health plan^(M6 and A11)^	No modifications	*C6 has had the health plan for two years now. The doctor comes to the house, and physiotherapy too, so there’s no need to take her out. (M6)* *Even with the pandemic, the appointments continued. That was great. Having a plan makes a difference at times like these, doesn’t it? (A11)*	
Ease due to the possibility of dedicating oneself to care^(M1, M8)^	No modifications	**Pre-pandemic context**	**Trans-pandemic context**
		*Yeah, no problem. Since I don’t work, I stay home because of C1. (M1)*	*I take care of him, which makes things much easier, so I do everything I can for him. (M1)*
Difficulty in managing multiple health appointments^(M13)^	No modifications	*I have to organize my schedule around C13’s therapies. It’s very demanding for me. (M13)*	*The only problem is getting her medical care during this pandemic. Without it, her development will be completely delayed. (M13)*
Difficulty in managing multiple health appointments^(M1)^	Improvement	*It’s a lot of doctors, you know, a lot of little things, and I, as a first-time mother, find it more complicated [...] he goes to many professionals. (M1)*	*I’m always taking him to the doctor. The good thing is that it’s nothing like it was before. I take him mostly for routine checkups and when his bronchitis flares up. (M1)*

### Parental Mutuality

According to FMSF, parental mutuality refers to the family’s beliefs regarding the extent to which they share or diverge in their perspectives on caring for their children^(8)^. In this study, there was no worsening in this dimension. Most families demonstrated alignment among family members in decision-making and care for CCC. Even in the face of contextual change, caregivers living with their partners maintained constant dialogue and shared decisions, balancing children’s demands.

Some reports highlighted the maintenance of a perception of normality and feelings of love as factors that strengthened care. In contrast, others revealed difficulty accepting children’s health condition or lack of support from the spouse in both contexts of the study. An improvement in family perspective was identified in the statements of F4 and M7, who reported that the COVID-19 pandemic increased family interaction and unity due to the longer time spent together at home. Chart 4 presents a synthesis of these perspectives.

**Chart 4:** Parental mutuality – Belo Horizonte, MG, Brazil, 2025.

Parental mutuality			
Family perspective	Pattern of perspective variation	Empirical data	
		Pre-pandemic context	Trans-pandemic context
Union and sharing of caregiving tasks^(M2, M3, F4, M6, M7, M10, M13, F15, C20, C21)^	No modifications	*What I have most in common with my husband is our concern for the C3. In this respect, we are very close and similar. (M3)*	*Thank God, we have no disagreements. We are very united in taking care of C3. (M3)*
Dialogue and shared decisions^(M3, M7, F15, M16 and M24)^	No modifications	*We maintain a dialogue about everything involving C16 and discuss all decisions. (M16)*	*My husband and I talk a lot, we make decisions together, even about not receiving visitors during the pandemic. (M16)*
Lack of dialogue and differing perspectives on care^(M14)^	No modifications	*My husband doesn’t help. We don’t talk about it either, I end up letting it go. We already have enough problems. (M14)*	*My husband doesn’t worry. I can’t even ask him for help. I can’t talk to him about it, so I handle things myself. (M14)*
Perspective of normality regarding children^(M3 and M23)^	No modifications	*My husband and I think alike. We have to treat C23’s life like any other normal child. (M23)*	*We treat her the same as our other child. For us, she’s a normal child. (M23)*
Non-acceptance of children’s health conditions^(M8)^	No modifications	*My husband doesn’t accept it at all. I don’t accept it either, but we accept that it was God who allowed this. (M8)*	*My husband has also been feeling a bit overwhelmed, just like me, because it’s a difficult thing to accept... we don’t accept it. (M8)*
A similar perspective on children’s education^(M7, F15 and M18)^	No modifications	*My vision regarding his education for the future is the same as my husband’s. (M18)*	*We value his upbringing very much, we teach him to be polite every day. (M18)*
Love^(M8 and M10)^	No modifications	*Her father and I both have a lot of affection for her. We love her very much. (M10)*	*We have a lot of love and, therefore, we take very good care of her together. (M10)*
Coexistence and strengthening of family ties^(F4 and M7)^	Improvement	**Trans-pandemic context**	
		*We had more dialogue and companionship at home. The pandemic brought us much closer together because of the increased time spent together. (F4)* *The pandemic gave us the opportunity to be more united, together, talk more about things and take care of C7. (M7)*	

## DISCUSSION

Data analysis made it possible to understand families’ perspectives regarding their children and their health conditions in the pre- and trans-pandemic contexts of COVID-19. In summary, the set of information produced allows recognition that the pandemic, as a contextual change, generated implications for the perspectives of families of CCC.

According to FMSF, the family’s view of children and their health condition significantly impacts care management^(8)^, since care actions directed toward CCC depend on how these children are perceived by their families. Thus, each dimension of the family perspective (child identity, illness view, management mindset, and parental mutuality) is closely interconnected and constitutes the family basis for caring for CCC^(8)^.

The interconnections among the dimensions of family perspective, presented in Figure 1, demonstrate how this perception guided care management over time. In the pre-pandemic context, the family perspective focused on the strengths of CCC (child identity) justified care actions directed toward health maintenance and promotion of child development (management mindset). By recognizing the developmental progress of their children, as well as the abilities they were capable of acquiring, families directed their efforts toward ensuring that these advances remained continuous. In the trans-pandemic context, although the predominant family view was not centered on the strengths of CCC, recognition of positive aspects sustained family efforts to maintain educational and rehabilitation activities at home after their interruption by healthcare and educational services.

It is important to emphasize that, in the trans-pandemic context, the need to maintain rehabilitation and educational activities at home was recognized by families as a difficulty (management mindset). However, it represented an active response to the suspension of elective appointments and school activities, reflecting their commitment to ensuring continuity of developmental stimulation for their children. These findings highlight the importance of family competence in maintaining care during situations of fragility in healthcare service provision and reaffirm the relevance of nurses’ participation in the continuous development of these competencies, so that families, as a constant presence in children’s lives, can act to ensure continuity of care even in unexpected contexts. Nevertheless, a limit to families’ capacity to maintain these activities should be considered, since the needs of CCC change over time and may no longer be adequately met in home environments.

Thus, in professional practice, nurses cannot dispense with interventions directed toward the needs of CCC, since early childhood is a critical period for physical, cognitive, and emotional development. During this phase, important brain formations and acquisitions occur that support future, more complex abilities^(20)^. Therefore, it is essential to ensure specialized healthcare services and qualified professional practice, even in adverse contexts such as the COVID-19 pandemic, ensuring that family care has a complementary rather than substitutive role in relation to professional care.

This study demonstrated that the COVID-19 pandemic was understood by families as an aggravating factor that directly threatened the lives of children with preexisting chronic conditions. This perception reinforced the view of children (child identity) as vulnerable and expanded awareness regarding the severity of their health condition (illness view). In response, families incorporated or intensified preventive measures such as rigorous hygiene practices, mask use, and social distancing — challenging practices in daily life, but maintained as care management strategies (management mindset). Such adaptations reveal families’ capacity for reorganization in the face of pressures imposed by the health crisis and highlight the need for continuous professional support to sustain care quality.

It is worth considering that, during data production in the pandemic context, strict social distancing measures were still in force and uncertainties persisted regarding the disease, its consequences, and the possibility of a vaccine. This scenario may have influenced families’ views regarding children and their health condition, since the experienced context has the potential to directly modify such perceptions^(8)^. Although most children infected with SARS-CoV-2 presented mild symptoms^(21)^, severe cases, hospitalizations, and deaths occurred predominantly among those with previous comorbidities^(21)^, justifying caregivers’ concerns and the adoption or intensification of preventive measures to protect their CCC. Furthermore, information disseminated by the media and social networks during the pandemic^(22)^ may also have contributed to reinforcing families’ perceptions regarding children’s vulnerability and the severity of their health conditions.

In both contexts of this investigation, some families highlighted a perception of normality regarding their children (child identity). Although this was not the predominant perspective, normalization was emphasized by families whose children presented chronic conditions with less complex care demands. Even while recognizing the vulnerabilities imposed by the chronic condition and the need for continuous care, families also expressed their perception of normality. This finding suggests that families are moving toward effective care management, and this perspective may represent a resource used to care for children without limiting them to the presence of a chronic condition.

Regarding management mindset, facilitating aspects of care common to both analyzed contexts were identified, such as the possibility of exclusive dedication to children and access to private health insurance. These perceptions appear to reflect the family’s perspective regarding children (child identity), recognizing the need for continuous care. Full-time dedication to care emerges as a strategy to meet multiple demands, while access to private health insurance played different roles according to the period: in the pre-pandemic context, it facilitated home care, reducing travel amid family overload; in the trans-pandemic context, it contributed to continuity of care even with the suspension of in-person services, demonstrating that although it was not a facilitator produced by the context itself, its function was directly influenced by it.

A systematic review on the health of parents of CCC indicates that exclusive dedication to care is a facilitating factor due to the intense routine of home care and specialized appointments, which is often incompatible with any paid employment for the primary caregiver^(23)^. However, this centralization of demands on the primary caregiver may increase caregiver burden. This study demonstrated that caregivers were predominantly female and that most mothers did not work outside the home, dedicating themselves exclusively to the household and children, in agreement with findings from the literature indicating that women stop working to remain at home due to the demands imposed by chronic conditions in childhood^(24)^.

It is known that, throughout the COVID-19 pandemic, social distancing measures led to a reduction in support networks for families of CCC, making continuity of care for CCC even more dependent on primary caregivers^(25)^. However, as evidenced in this study, increased family coexistence during the pandemic favored greater family cohesion, strengthening emotional bonds and the perception of unity in caregiving. Parental mutuality, therefore, contributes to more stable and adaptive management, since it minimizes conflicts and distributes responsibilities, reducing individual burden and promoting greater alignment in care actions^(26)^.

It is observed that the context experienced by families of CCC, especially during the COVID-19 pandemic, directly impacted care management, intensifying demands, highlighting vulnerabilities, and requiring adaptation of family routines. These findings reinforce the need for attentive and specialized professional care, together with the development of public policies and strategies that effectively support families in emergency situations.

Some limitations of this study should be acknowledged. Data production in the trans-pandemic context occurred during a specific moment of the COVID-19 pandemic, marked by high uncertainty, absence of immunization, and strict restrictions, which may have influenced family perceptions regarding children and care, limiting the extrapolation of the findings to other periods of the health crisis. Studies involving CCC with greater complexity, conducted in other countries or in distinct sociocultural contexts, may present different results due to the various strategies used to confront the pandemic, cultural specificities, and adopted healthcare policies.

## CONCLUSION

The findings of this study demonstrate that the family perspective regarding CCC guides care management over time and is sensitive to contextual changes. During the pre-pandemic period, a perspective anchored in children’s strengths supported practices directed toward the promotion of child development and health maintenance. In the trans-pandemic context, COVID-19 was identified as an additional aggravating factor to the chronic condition, being perceived as a direct threat to life and contributing to a family perspective focused on children’s fragility. This resulted in increased surveillance and incorporation of new hygiene demands into the family routine. Even so, recognition of children’s positive aspects remained a mobilizing element for care, favoring the maintenance of educational and rehabilitation activities within home environments by the family. These findings reinforce the dynamic nature of family perspective and its centrality in care management.

In light of these findings, the study offers implications for nursing practice by highlighting the need for nurses to recognize family perspective as a central element in the assessment and planning of care for CCC, especially in the face of changes in their life contexts, such as health crises. The use of theoretical frameworks such as FMSF may assist professionals in identifying how caregivers perceive children and organize care in daily life, favoring family-centered interventions. In the field of public policies, the findings reinforce the importance of maintaining continuity of care, strengthening support networks, and organizing care responses that consider the specific demands of families in crisis situations, especially when such situations alter the provision of services required by children. Future research is needed to deepen understanding of family perspective in other contexts and over time.

## DATA AVAILABILITY

The entire dataset supporting the results of this study is available upon request to the corresponding author.
