# Supplementary material for: Family perspective on caring for children with chronic conditions in the pre- and trans-pandemic contexts of COVID-19
Source: Rev Esc Enferm USP. 2026 Jul 24;60:e20250448. doi: 10.1590/1980-220X-REEUSP-2025-0448en (PMC13399439; doi:10.1590/1980-220X-REEUSP-2025-0448en)
Supplement: Translation

# Translation: Perspectiva familiar sobre cuidar de crianças com condições crônicas nos contextos pré- e transpandemia de COVID-19

**DOI:** 10.1590/1980-220X-REEUSP-2025-0448pt

**Authors:** Nayara Luiza Henriques, Maísa Mara Lopes Macêdo Breves, Melissa Joice de Abreu Felizardo, Gabriel Lucas Santos Galdino, Samylla Alves de Almeida, Elysângela Dittz Duarte

**Affiliations:** 1 Universidade Federal de Minas Gerais, Escola de Enfermagem, Departamento de Enfermagem Materno-Infantil e Saúde Pública, Belo Horizonte, MG, Brasil.

**Keywords:** Doença Crônica, Cuidado da Criança, Família, COVID-19, Cuidados de Enfermagem

## Abstract

**Objetivo:**

Compreender a perspectiva das famílias sobre cuidar de crianças com condições crônicas nos contextos pré- e transpandemia de COVID-19 à luz do *Family Management Style Framework*.

**Métodos:**

Estudo qualitativo longitudinal com 24 cuidadores familiares de crianças de 2 a 4 anos. Os dados foram obtidos por questionário socioeconômico e entrevistas semiestruturadas em duas etapas: pré-pandemia (outubro/2019 a maio/2020) e transpandemia (junho/2020 a janeiro/2021), sendo submetidos à análise temática guiada pelo referencial teórico.

**Resultados:**

A perspectiva familiar orientou o manejo do cuidado ao longo do tempo e foi modificada pela pandemia. No contexto pré-pandemia, esteve centrada nas potencialidades da criança, direcionando ações de promoção do desenvolvimento e manutenção da saúde. No contexto transpandemia, a perspectiva teve foco nas fragilidades e vulnerabilidades da criança, associadas à percepção de ameaça da COVID-19, resultando na intensificação da vigilância e dos cuidados de higiene.

**Conclusão:**

A perspectiva familiar orienta o manejo do cuidado e é sensível às mudanças contextuais, oferecendo subsídios para a qualificação da prática profissional em contextos de crise sanitária.

## INTRODUÇÃO

A incorporação de novas tecnologias em saúde contribuiu para a crescente especialização dos cuidados materno-infantis^(1)^, repercutindo na redução das taxas de mortalidade infantil e no aumento de crianças vivendo com alguma condição crônica^(2)^. Condições crônicas na infância são aquelas que possuem origem congênita ou adquirida, com base biológica, psicológica ou cognitiva, que resultam em limitações funcionais permanentes ou temporárias^(3)^.

No âmbito domiciliar, as famílias são protagonistas do cuidado das crianças com condições crônicas (CCCs)^(4)^. Elas convivem com o constante desafio de adequar à rotina a execução dos cuidados essenciais para a manutenção da saúde das crianças, os quais incluem desde a alimentação e higiene até o manuseio de dispositivos tecnológicos complexos^(5)^. Além das tarefas domiciliares, os cuidadores frequentemente necessitam mobilizar recursos para comparecer regularmente às consultas especializadas e reorganizar a vivência familiar em casos de hospitalizações^(6)^. Tal conjunto de responsabilidades, inerentes ao curso de vida dessas crianças, constrói um cotidiano caracterizado pela sobrecarga e pelo estresse familiar^(7)^.

A magnitude dessa questão é ainda potencializada pelo contexto em que a família se insere. Segundo o *Family Management Style Framework* (FMSF)^(8)^, modelo teórico que auxilia na compreensão do manejo familiar da condição crônica na infância, o contexto constitui uma dimensão central do cuidado familiar, capaz de modificar as condições de vida da família e a maneira como ela percebe e gerencia o cuidado^(8)^.

Uma mudança contextual repentina foi vivenciada globalmente com o advento da pandemia de *Coronavirus Disease 2019* (COVID-19) em março de 2020^(9)^. Em razão do elevado potencial de disseminação do vírus, decretos governamentais instituíram medidas de distanciamento social, que resultaram na suspensão de atendimentos eletivos em saúde e no fechamento de serviços, como escolas e creches, além da adoção de medidas de proteção, como o uso de máscaras e a intensificação da higiene das mãos^(9)^. As implicações dessa alteração contextual afetaram a vida de todas as pessoas, especialmente das famílias de CCCs. Além de realizarem os cuidados de rotina demandados pelas crianças, incorporaram novas medidas de higiene e vivenciaram a restrição da convivência social, bem como a suspensão dos acompanhamentos em saúde e das atividades escolares. Desse modo, o contexto de crise sanitária impôs demandas adicionais às famílias, que já possuíam sobrecarga de tarefas, ao mesmo tempo em que reduziu suas redes de apoio^(10)^.

Diante das mudanças substanciais no cuidado das CCCs promovidas pela pandemia de COVID-19, sobretudo quando comparadas com o momento anterior à pandemia, parte-se do pressuposto de que a família modificou a sua perspectiva sobre a criança e sua condição de saúde, o que, por sua vez, repercutiu diretamente no manejo familiar do cuidado. Isso porque as ações de cuidado direcionadas às CCCs, que são as respostas ativas da família diante das suas necessidades, dependem de como elas são reconhecidas e vistas por suas famílias^(8)^. Assim, a perspectiva familiar sobre as capacidades e necessidades das CCCs molda diretamente a maneira como o cuidado é realizado^(8)^.

Observa-se um número crescente de estudos que se concentram na sobrecarga e no impacto emocional que o cuidado das CCCs pode gerar. Entretanto, a literatura disponível tem se dedicado predominantemente a esses aspectos, conferindo menor ênfase à análise da perspectiva das famílias sobre o cuidado em contextos distintos e marcados por mudanças abruptas, como a pandemia de COVID-19. Ademais, embora os estudos desenvolvidos durante a pandemia abordassem as transformações ocorridas no cuidado familiar, eles se basearam, em sua maioria, em dados coletados após o estabelecimento da crise sanitária, o que limita a análise da perspectiva familiar ao longo do tempo e diante da mudança contextual, lacuna contemplada por esta investigação.

Diante desse cenário, definiu-se a seguinte questão de pesquisa: qual é a perspectiva das famílias sobre cuidar de uma CCC nos contextos pré- e transpandemia de COVID-19? Para responder a esta pergunta, o estudo tem como objetivo compreender a perspectiva das famílias sobre cuidar de CCCs nos contextos pré- e transpandemia de COVID-19 à luz do FMSF.

## MÉTODO

### Desenho do Estudo

Trata-se de estudo qualitativo longitudinal^(11)^, cuja elaboração atendeu às recomendações de transparência descritas na ferramenta de consenso denominada como Critérios Consolidados de Relato de Pesquisa Qualitativa (COREQ), versão brasileira^(12)^.

### Referencial Teórico-Metodológico

Para esta investigação, adotou-se como referencial teórico o FMSF^(8)^, modelo desenvolvido pelas enfermeiras Kathleen Knafl e Janet Deatrick para compreender como as famílias percebem e respondem ativamente às demandas de cuidado de uma CCC considerando o contexto em que estão inseridas. Como referencial metodológico, adotou-se a análise temática proposta por Braun e Clarke^(13)^, que possibilita a interpretação de dados da pesquisa a partir de categorias previamente definidas e fundamentadas no referencial teórico. Neste estudo, a análise concentrou-se em um dos componentes do FMSF, denominado “definição da situação”, que permite apreender a perspectiva da família sobre o cuidado da CCC.

### Participantes

Participaram 24 cuidadores familiares de CCCs com idade entre 2 e 4 anos, egressas de duas Unidades de Terapia Intensiva Neonatal (UTINs) vinculadas a um hospital filantrópico e a um hospital federal, ambos localizados em uma capital da região Sudeste do Brasil. As instituições foram escolhidas por serem referências nacionais do Sistema Único de Saúde no cuidado materno-infantil.

### Definição da Amostra e Critérios de Seleção

A definição da amostra foi intencional e não probabilística, baseada em critérios de elegibilidade. Foram incluídos cuidadores familiares responsáveis pelo cuidado de CCCs, com idade entre 2 e 4 anos, considerando que as alterações no desenvolvimento neuropsicomotor se tornam mais evidentes nessa faixa etária^(14)^. Os cuidadores deveriam ter idade igual ou superior a 18 anos, apresentar viabilidade para contato telefônico e residir no mesmo domicílio da criança, uma vez que foram considerados informantes-chave da dinâmica familiar envolvida na garantia do cuidado da CCC no ambiente domiciliar, sendo, portanto, fundamental que compartilhassem o mesmo espaço de convivência^(15)^. Foram excluídos cuidadores com comprometimentos de comunicação ou alterações psicológicas e/ou psiquiátricas que inviabilizassem a coleta de dados, bem como aqueles que não responderam após três tentativas consecutivas de contato telefônico.

### Coleta de Dados

O estudo foi realizado em duas etapas, correspondentes aos dois contextos investigados: etapa 1 (contexto pré-pandemia da COVID-19), que abrangeu o período de outubro de 2019 a maio de 2020, durante o qual ocorreu a primeira fase de produção de dados com as famílias, antecedendo a declaração da COVID-19 como pandemia pela Organização Mundial da Saúde em 11 de março de 2020^(9)^; e etapa 2 (contexto durante a pandemia ou transpandemia), referente ao período de junho de 2020 a janeiro de 2021, considerado neste estudo como o intervalo em que se deu a produção de dados no contexto da pandemia, caracterizando o transcurso dessa situação inédita no mundo^(16)^.

A identificação dos participantes ocorreu na primeira etapa do estudo (contexto pré-pandemia). Para isso, foram analisados todos os prontuários de crianças que receberam alta da UTIN entre dezembro de 2016 e dezembro de 2017, com o intuito de incluir crianças com idade entre 2 e 4 anos.

A partir da análise de prontuários, foram identificados 1.115 cuidadores, sendo 852 provenientes do hospital filantrópico e 263 do hospital federal. Entre outubro de 2019 e maio de 2020, todos foram contatados por telefone para a aplicação do Questionário para Identificação de Crianças com Condições Crônicas – Revisado (QuICCC-R), versão brasileira, instrumento utilizado para rastrear as CCCs^(17)^. Do total de cuidadores identificados, não foi possível estabelecer contato com 829, devido a números inexistentes ou desatualizados. Assim, 286 cuidadores foram contatados. Desses, dez se recusaram a participar; cinco informaram que suas crianças haviam falecido; e 218 possuíam crianças que não apresentavam condições crônicas segundo o QuICCC-R, resultando em 53 cuidadores participantes da coleta de dados da primeira etapa (pré-pandemia).

Com o advento da pandemia de COVID-19, o estudo foi ampliado, com a anuência do Comitê de Ética, incluindo uma segunda etapa que se referiu ao contexto transpandemia. Foram feitas tentativas de contato com os mesmos 53 cuidadores que participaram da primeira etapa. Desses, nove não possuíam o mesmo número de telefone; 13 não responderam ao contato após três tentativas; e quatro não aceitaram participar. Restaram, assim, 27 cuidadores, dos quais três fizeram parte de um estudo piloto para a validação do roteiro das entrevistas e não foram incluídos na análise de dados. Assim, a população definida para este estudo foi composta por participantes que estiveram presentes em ambas etapas, totalizando 24 cuidadores. Ressalta-se, ainda, que, nos dois momentos da coleta de dados, considerou-se respondente o mesmo familiar.

Na primeira etapa do estudo, após a confirmação da condição crônica da criança, as famílias foram convidadas a participar do estudo, e as entrevistas foram agendadas. Inicialmente, elas foram realizadas no domicílio das famílias. No entanto, devido à dificuldade de deslocamento das pesquisadoras para o interior de Minas Gerais, onde a maioria das famílias residia, e com a aprovação do Comitê de Ética, as entrevistas passaram a ser realizadas por telefone. Na segunda etapa, referente ao contexto transpandemia, considerando as normas de segurança implementadas durante o contexto pandêmico, todas as entrevistas foram realizadas por telefone e gravadas após o consentimento verbal do participante.

Em ambas as etapas, aplicou-se um questionário socioeconômico para coletar informações sobre a criança (sexo, idade gestacional e cronológica e diagnóstico) e o familiar (grau de parentesco, idade, etnia, escolaridade, situação conjugal, ocupação e renda). Na sequência, utilizou-se um roteiro semiestruturado, elaborado com base no FSMF^(8)^. Em ambas as fases, o foco das entrevistas foi a perspectiva familiar sobre o cuidado das CCCs. As entrevistas foram conduzidas por duas pesquisadoras, mestres e doutorandas em enfermagem, com experiência em entrevistas qualitativas e sem vínculo prévio com as famílias.

### Análise e Tratamento de Dados

As entrevistas foram transcritas na íntegra e submetidas à análise temática do tipo dedutiva guiada pelo referencial teórico do FMSF. Para isso, foi adotado o processo analítico proposto por Braun e Clarke^(13)^, composto por seis fases: 1) Familiarização com os dados por meio da transcrição e leitura das entrevistas; 2) Geração inicial de códigos preliminares com base no referencial teórico, abrangendo o componente “definição da situação” do FMSF e suas respectivas dimensões conceituais: identidade da criança; visão da condição; mentalidade de manejo; e mutualidade entre os pais; 3) Elaboração de resumos dos trechos codificados das entrevistas e reconhecimento de padrões; 4) Revisão dos padrões identificados para garantir sua distinção e consistência;5) Elaboração de definições para cada padrão/tema identificado; 6) Descrição e análise dos resultados, relacionando-os ao referencial teórico e ao objetivo do estudo.

### Aspectos Éticos

A pesquisa foi desenvolvida em conformidade com as Resoluções n^o^ 466/2012 e n^o^ 580/2018 do Conselho Nacional de Saúde^(18,19)^, sendo aprovada pelo Comitê de Ética em Pesquisa sob o Parecer nº 3.508.414/ 2019 e a Emenda nº 4.331.516/2020. Os participantes assinaram o Termo de Consentimento Livre e Esclarecido em duas vias. Visando assegurar o sigilo e anonimato, seus nomes foram substituídos nas entrevistas por códigos alfanuméricos utilizando-se as letras M (“mãe”), P (“pai”), T (“tia”) e C (“criança”). As letras foram seguidas pelo número correspondente à ordem de realização das entrevistas (ex.: M4, P3, T1).

## RESULTADOS

### Caracterização das Famílias e Crianças com Condições Crônicas

A maioria dos cuidadores participantes eram mães (n = 22). Apenas uma entrevista foi realizada com o pai e outra com a tia da CCC. A idade média dos respondentes foi de 34 anos, sendo a mínima 19 e a máxima 54 anos. A maioria dos participantes se declarou casada (n = 12), parda (n = 15), com ensino superior completo (n = 8), residindo com o companheiro (n = 20) no interior de Minas Gerais (n = 16). A renda familiar referida foi entre um e dois salários mínimos (n = 11), que, à época, correspondia ao valor de R$1.100,00. Sete cuidadores referiram renda inferior a um salário mínimo. Das mães participantes, 14 não estavam inseridas no mercado de trabalho e se dedicavam ao cuidado da CCC. No contexto transpandemia, 17 participantes relataram ter recebido algum tipo de auxílio financeiro. No que se refere às CCCs, a maioria era do sexo masculino (n = 15), com idade média de 2 anos e 8 meses. A média da idade gestacional ao nascimento foi de 31,8 semanas. Os diagnósticos médicos predominantes foram de natureza neurológica (n = 10).

### Perspectiva das Famílias Sobre o Cuidado das Crianças com Condições Crônicas

A análise de dados foi direcionada à compreensão da perspectiva das famílias sobre o cuidado das CCCs. Para isso, utilizou-se o componente conceitual “definição da situação” do FMSF, que permite compreender como os cuidadores percebem a criança e a sua condição crônica, bem como a influência dessa percepção na gestão do cuidado. Esse componente abrange quatro dimensões exploradas neste estudo: identidade da criança, visão da doença, mentalidade de manejo e mutualidade parental.

Realizou-se uma comparação das informações produzidas nos contextos pré- e transpandemia de COVID-19 para identificar padrões e variações nas perspectivas das famílias ao longo do tempo, evidenciando situações de melhora, piora ou ausência de modificações. Essa síntese está representada na Figura 1, na qual cada dimensão do componente “definição da situação” é destacado, sendo que as linhas pontilhadas indicam a interconexão entre elas conforme o discurso dos participantes. Após a figura, cada uma das dimensões será apresentada detalhadamente.

**Figura 1:** Perspectiva da família sobre o cuidado da criança com condição crônica nos contextos pré- e transpandemia de COVID-19 – Belo Horizonte, MG, Brasil, 2025.

### Identidade da Criança

Segundo o FMSF, a identidade da criança refere-se à perspectiva da família sobre a CCC, considerando o reconhecimento de suas potencialidades e fragilidades^(8)^. Neste estudo, no contexto pré-pandemia, a maioria das famílias enfatizou as potencialidades das crianças, como habilidades adquiridas (ficar em pé, falar, correr, se alimentar sozinha) e características positivas (independência, inteligência, boa memória e facilidade em aprender). Já no contexto transpandemia, a perspectiva se modificou e teve foco nas fragilidades, sendo relacionada especialmente à imunidade baixa, ao comprometimento pulmonar ou ao reconhecimento da CCC enquanto susceptível ao adoecimento por COVID-19. Dessa forma, os padrões de piora na perspectiva foram os mais recorrentes diante da mudança contextual.

A ausência de modificações na perspectiva familiar evidenciou-se no reconhecimento de que as crianças demandam cuidados continuados e vigilância constante em ambos os contextos estudados. Ou seja, o foco se manteve nas vulnerabilidades da criança antes e durante a pandemia de COVID-19. Essa percepção foi mais frequente entre famílias de crianças com condições de saúde que exigiam cuidados mais complexos. Por outro lado, entre aquelas cujas crianças apresentavam demandas de cuidado menos complexas, a perspectiva manteve-se centrada na normalidade da criança ao longo dos dois momentos investigados.

Foi identificada apenas uma situação de melhora, relatada por M1, cuja criança apresentava, no contexto pré-pandemia, dificuldades para andar devido a pé torto congênito. Com a resolução do problema ao longo do tempo, houve mudança positiva na percepção da família relacionada à melhora do quadro de saúde da criança, não ao contexto pandêmico. O Quadro 1 apresenta as informações relativas à perspectiva das famílias sobre a CCC. Na primeira coluna, encontram-se as perspectivas familiares acompanhadas da menção das famílias que as relataram; na segunda, encontra-se o padrão de variação observado (melhora, piora ou ausência de modificação); e na terceira, encontram-se exemplos de enunciados que ilustram a perspectiva ao longo do tempo.

**Quadro 1:** Identidade da criança – Belo Horizonte, MG, Brasil, 2025.

Identidade da criança			
Perspectiva da família	Padrão de variação da perspectiva	Dados empíricos	
		Contexto pré-pandemia	Contexto transpandemia
Susceptibilidade da criança ao adoecimento no contexto transpandemia^(M1, M2, M3, M5, M6, M8, M9, M14, M16, M17, M18, M21 e M22)^	Piora	*Ele é um menino tranquilo, não é daqueles meninos de viver no hospital, não. Na saúde dele, ele é tranquilo [...] e ele já consegue falar e fica em pé! (M1)*	*Eu tenho medo mesmo é dele pegar o corona. Por ele ter bronquite, eu acho que é mais fácil pra ele pegar. A imunidade dele é mais baixa, né? Ele é do grupo de risco. (M1)*
Necessidade de cuidados continuados em ambos os contextos^(M8, M12 e M13)^	Sem modificações	*A C12 exige atenção 24 horas. Pra tudo que ela vai fazer, ela depende de uma pessoa. (M12)*	*Ela é uma criança que precisa de cuidado o tempo todo, pra tudo: pra comer, pra tomar banho, pra se locomover. A parte motora dela é toda comprometida. (M12)*
Visão de normalidade em ambos os contextos^(M3, P4, M6, M7, T11, P15, M20, M23, M24)^	Sem modificações	*A gente já vê a C15 como uma criança normal, porque ela é super inteligente. O desenvolvimento dela é bom. (P15)*	*O desenvolvimento dela é excelente. Na pandemia, graças a Deus, ela está se desenvolvendo bem. Tudo normal. (P15)*
Capacidade para o desenvolvimento de atividades^(M2, M3, M4, M5, M6, M7, M9, M10, T11, M12, M13 e M14)^	Sem modificações	*Graças a Deus, ela melhorou muito. Hoje em dia, ela já fica em pé sozinha; ela dá uns passinhos; a gente vê o avanço dela. (M6)*	*Na fala, eu já tenho conseguido fazer uns resultados. Ela já fala “mamãe”, “papai”, “aqui”, “opa”, umas coisinhas monossilábicas. (M6)*
Capacidade para o desenvolvimento de atividades^(M1)^	Melhora	*O C1 cai direto e eu tenho medo dele cair, porque ele bate direto o rosto no chão e eu fico com medo dele bater a cabeça. (M1)*	*Ele não cai tanto igual antes. Os pezinhos dele eram um pouco mais pra dentro, aí arrumaram e ele parou de cair. (M1)*

### Visão da Doença

Segundo o FMSF, a visão da doença diz respeito à compreensão da família sobre a condição de saúde da criança^(8)^. Neste estudo, identificou-se que, no período pré-pandemia, as famílias reconheciam a gravidade do estado de saúde das CCCs. Com o advento da pandemia, passaram a perceber que a infecção pelo vírus SARS-CoV-2 representava um risco adicional, configurando ameaça direta à vida de suas crianças e potencializando a perspectiva de gravidade do estado de saúde delas. Assim, apenas situações de piora na visão da doença foram identificadas, conforme exemplificado no Quadro 2.

**Quadro 2:** Visão da doença – Belo Horizonte, MG, Brasil, 2025.

Visão da doença			
Perspectiva da família	Padrão de variação da perspectiva	Dados empíricos	
		Contextopré-pandemia	Contexto transpandemia
Percepção da gravidade do quadro clínico da criança associada ao risco potencial de agravamento pela COVID-19^(M1, M2, M3, M5, M6, M8, M9, M14, M16, M17, M18, M21, M22 e M24)^	Piora	*O C24 tem uma cardiopatia congênita grave, né? Então, fazemos os acompanhamentos direitinho. (M24)*	*Se ele pegar o vírus, pode piorar ainda mais a cardiopatia dele; não sei se escapa. (M24)*

### Mentalidade de Manejo

Segundo o FMSF, a mentalidade de manejo compreende a perspectiva da família sobre facilidades e/ou dificuldades em realizar o regime de cuidado da CCC^(8)^. Ao comparar os discursos das famílias em ambos os contextos investigados, observa-se que padrões de piora em suas perspectivas são os mais frequentes. Eles se caracterizam pelas dificuldades mencionadas em relação ao contexto da pandemia de COVID-19, como intensificação dos cuidados de higiene para contenção da doença e restrição da família ao domicílio, que levaram a repercussões emocionais como ansiedade e estresse. A interrupção das terapias de reabilitação e de educação durante a pandemia culminaram na necessidade de a família assumir e realizar essas atividades no domicílio, situações que levaram a uma pior perspectiva sobre o manejo do cuidado. M13 relata uma dificuldade que manteve em ambos os contextos do estudo relacionada às múltiplas consultas que sua criança frequentava. No contexto pré-pandemia, o desafio da mãe era se organizar para estar presente nos atendimentos. Já no contexto transpandemia, a dificuldade esteve relacionada à interrupção dos atendimentos.

Em relação às facilidades para o cuidado, que permaneceram inalteradas ao longo do tempo, destacaram-se a continuidade das terapias e dos atendimentos em saúde mesmo durante a pandemia, a possibilidade de dedicação exclusiva aos filhos e a presença de plano de saúde. Apenas uma situação de melhora foi relatada por M1, associada à redução do número de consultas em decorrência da evolução clínica da criança, o que facilitou o manejo do cuidado. Nesse caso, a melhora refletiu a condição de saúde da criança, não o contexto pandêmico. O Quadro 3 exemplifica as informações apresentadas.

**Quadro 3:** Mentalidade de manejo – Belo Horizonte, MG, Brasil, 2025.

Mentalidade de manejo			
Perspectiva da família	Padrão de variação da perspectiva	Dados empíricos	
		Contexto transpandemia de COVID-19	
Dificuldade em relação à interrupção dos serviços de saúde e educação^(M2, M5, M6, M7, M10, T11, M16 e M22)^	Piora	*Tá sendo assim: uma correria, né? Mais estressante, porque não tem escola, né? Então, eles ficam em casa mais ansiosos. (M7)* *A gente teve que parar com a aula de natação. O ambulatório do hospital não teve consulta. Aqui na APAE, o médico também parou, então, assim, o cuidado ficou difícil. (M22)*	
Dificuldade devido à manutenção de atividades de reabilitação e de educação no domicílio^(M2, M5, M6, M7, M10, T11, M16 e M22)^	Piora	*Não está tendo esses atendimentos presenciais. Aí, no caso, está tendo fisioterapia online, né? Aí eles ligam, fazem chamada de vídeo, aí, no caso, eu que estou tendo que fazer fisioterapia. (M10)* *As atividades da escola vêm pra eu ensinar. Não é fácil, viu? (C6)*	
Dificuldade devido à intensificação dos cuidados de higiene^(M1, M3, M8, M9, M23 e M24)^	Piora	*Eu estou tendo mais cautela com o C1. Eu não saio tanto igual antes, estou sempre lavando as mãozinhas dele; quando eu saio, eu chego em casa e já troco a roupinha dele, já dou banho. Meu cuidado com ele dobrou. (M1)* *Eu passo pano em tudo todos os dias com água sanitária para desinfetar. Nas coisas da C8 eu borrifo álcool. Agora, na pandemia, é muito difícil manter os cuidados dela e esses cuidados, mas estou mantendo todos. (M8)*	
Dificuldade devido à restrição da criança e da família ao domicílio^(M1, M3, M7, M10, M13, M14, M17 e M19)^	Piora	*Ficar mais preso em casa é ruim, né? A gente acaba ficando mais estressado. Não pode sair direito, não pode ter liberdade. (M1)* *Ah, teve mudança, né? Porque a gente ficou todo mundo dentro de casa, principalmente pra mim. Pra mim, é muito cansativo e estressante. (M13)*	
Facilidade em relação à manutenção das terapias durante a pandemia^(M1, M2, M3, M6. M7, M9, T11)^	Sem modificações	*Graças a Deus, a equoterapia está funcionando. É a única atividade liberada na minha cidade. (M3)* *Eles fazem acompanhamento na APAE, e está sendo muito importante pra eles agora na pandemia. (M7)*	
Facilidade devido ao acesso da criança ao plano de saúde^(M6 e T11)^	Sem modificações	*A C6 tem convênio já tem dois anos. O médico vem em casa, fisioterapia também, então não precisa ficar saindo com ela. (M6)* *Mesmo com a pandemia, continuou os atendimentos. Isso foi bom demais. Ter um plano faz diferença nessas horas, né? (T11)*	
Facilidade devido à possibilidade de se dedicar ao cuidado^(M1, M8)^	Sem modificações	**Contexto pré-pandemia**	**Contexto transpandemia**
		*É, tranquilo. Como eu não trabalho, eu fico em casa por conta do C1. (M1)*	*Eu fico por conta dele, o que facilita demais, então eu faço tudo que eu posso por ele. (M1)*
Dificuldade em relação aos múltiplos acompanhamentos em saúde^(M13)^	Sem modificações	*Eu tenho que montar os meus horários de acordo com as terapias da C13. É muito puxado para mim. (M13)*	*O único problema é conseguir atendimento para ela nessa pandemia. Sem isso, o desenvolvimento dela fica todo atrasado. (M13)*
Dificuldade em relação aos múltiplos acompanhamentos em saúde^(M1)^	Melhora	*É muito médico, né, muita coisinha, e eu, mãe de primeira viagem, acho mais complicado [...] ele vai em muitos profissionais. (M1)*	*Estou sempre levando ele ao médico. O bom é que nem se compara a antes. Eu levo ele mais na consulta de rotina e quando a bronquite ataca. (M1)*

### Mutualidade Parental

Segundo o FMSF, a mutualidade parental diz respeito às crenças da família acerca do quanto possuem perspectivas compartilhadas ou divergentes sobre o cuidado de suas crianças^(8)^. Neste estudo, não houve piora nessa dimensão. A maioria das famílias apresentou alinhamento entre os membros na tomada de decisão e no cuidado das CCCs. Mesmo diante da mudança contextual, cuidadores que residiam com seus parceiros mantiveram diálogo constante e decisões compartilhadas, equilibrando as demandas da criança.

Alguns relatos destacaram a manutenção da visão de normalidade e do sentimento de amor como fator fortalecedor do cuidado. Em contrapartida, outros evidenciaram dificuldade de aceitação da condição de saúde da criança ou falta de apoio do cônjuge em ambos os contextos do estudo. Uma melhora na perspectiva familiar foi identificada nos discursos de P4 e M7, que afirmaram que a pandemia de COVID-19 aumentou a convivência e a união familiar devido ao maior tempo de permanência no domicílio juntos. O Quadro 4 apresenta a síntese dessas perspectivas.

**Quadro 4:** Mutualidade parental – Belo Horizonte, MG, Brasil, 2025.

Mutualidade parental			
Perspectiva da família	Padrão de variação da perspectiva	Dados empíricos	
		Contexto pré-pandemia	Contexto transpandemia
União e compartilhamento das tarefas de cuidado^(M2, M3, P4, M6, M7, M10, M13, P15, C20, C21)^	Sem modificações	*Em comum com o meu marido, o que mais tenho é o cuidado com o C3. Neste ponto, nós somos muito unidos e parecidos. (M3)*	*Graças a Deus, não temos nenhuma divergência. Somos muito unidos para cuidar do C3. (M3)*
Diálogo e decisões compartilhadas^(M3, M7, P15, M16 e M24)^	Sem modificações	*Nós mantemos um diálogo sobre tudo que envolve a C16 e discutimos todas as decisões. (M16)*	*Eu e meu marido conversamos muito, tomamos as decisões juntos, até sobre isso de não receber mais visitas na pandemia. (M16)*
Ausência de diálogo e perspectivas distintas sobre o cuidado^(M14)^	Sem modificações	*Meu marido não ajuda. Nós também não conversamos sobre isso, eu acabo deixando pra lá. Já temos problemas demais. (M14)*	*O meu marido não se preocupa. Eu não consigo nem cobrar ajuda dele. Eu não dou conta de conversar sobre isso com ele, então eu resolvo as coisas. (M14)*
Perspectiva de normalidade sobre a criança^(M3 e M23)^	Sem modificações	*Eu e meu marido pensamos iguais. A gente tem que levar a vida da C23 como a de outra criança normal. (M23)*	*Nós tratamos ela igual ao nosso outro filho. Para nós, é uma criança normal. (M23)*
Não aceitação da condição de saúde da criança^(M8)^	Sem modificações	*Meu marido não aceita de jeito nenhum. Eu também não aceito, mas a gente conforma que foi Deus que permitiu isso.(M8)*	*O meu marido também tem ficado meio oprimido, igualzinho a mim, porque é uma coisa difícil de se aceitar... a gente não aceita. (M8)*
Perspectiva semelhante sobre a educação da criança^(M7, P15 e M18)^	Sem modificações	*A minha visão com relação à educação dele para o futuro é a mesma do meu marido. (M18)*	*Prezamos muito pela educação dele, ensinamos ele a ser educado todos os dias. (M18)*
Amor^(M8 e M10)^	Sem modificações	*Eu e o pai dela, nós dois temos em comum muito carinho com ela. Amamos demais ela. (M10)*	*Temos muito amor e, por isso, cuidamos muito bem dela juntos. (M10)*
Convivência e estreitamento de laços familiares^(P4 e M7)^	Melhora	**Contexto transpandemia**	
		*A gente teve mais diálogo e companheirismo dentro de casa. A pandemia uniu muito a gente por conta da convivência maior. (P4)* *A pandemia nos proporcionou essa possibilidade de ficar mais unido, juntos, conversar mais sobre as coisas e cuidar do C7. (M7)*	

## DISCUSSÃO

A análise de dados permitiu compreender a perspectiva das famílias sobre suas crianças e sua condição de saúde no contexto pré- e transpandemia de COVID-19. Em síntese, o conjunto das informações produzidas permite reconhecer que a pandemia, enquanto uma mudança contextual, produziu implicações na perspectiva das famílias de CCCs.

Segundo o FMSF, a visão que a família tem sobre a criança e a sua condição de saúde impacta significativamente o manejo do cuidado^(8)^, pois as ações de cuidado direcionadas às CCCs dependem de como elas são vistas por suas famílias. Desse modo, cada uma das dimensões da perspectiva das famílias (identidade da criança, visão da doença, mentalidade de manejo e mutualidade parental) está intimamente ligada e é a base familiar para o cuidado familiar da CCC^(8)^.

As interconexões entre as dimensões da perspectiva familiar, apresentadas na Figura 1, evidenciam como essa percepção orientou o manejo do cuidado ao longo do tempo. No contexto pré-pandemia, a perspectiva familiar focalizada nas potencialidades das CCCs (identidade da criança) justificou as ações de cuidado voltadas à manutenção da saúde e à promoção do desenvolvimento infantil (mentalidade de manejo). Ao reconhecerem as evoluções no desenvolvimento de suas crianças, bem como as habilidades que elas são capazes de adquirir, as famílias direcionaram os seus esforços para assegurar que essas evoluções fossem contínuas. Já no contexto transpandemia, embora a visão predominante da família não estivesse centrada nas potencialidades das CCCs, o reconhecimento de aspectos positivos sustentou o esforço familiar em manter, no domicílio, atividades de ensino e reabilitação interrompidas pelos serviços de saúde e educação.

É importante destacar que, no contexto transpandemia, a necessidade de manutenção das atividades de reabilitação e de educação no domicílio foi reconhecida como uma dificuldade pelas famílias (mentalidade de manejo). Entretanto, foi uma resposta ativa à suspensão das consultas eletivas e atividades escolares, que reflete o seu empenho em assegurar a continuidade do estímulo ao desenvolvimento das suas crianças. Essas informações destacam a importância da competência familiar para a manutenção do cuidado em situações de fragilidade na oferta de serviços de saúde e reafirmam a relevância da participação do enfermeiro no desenvolvimento constante dessas competências, de modo que as famílias, sendo uma constante na vida das crianças, consigam atuar para garantir a continuidade do cuidado, mesmo em contextos inesperados. Todavia, deve-se considerar um limite para a capacidade das famílias manterem essas atividades, uma vez que as necessidades das CCCs se modificam com o passar do tempo, podendo não serem mais atendidas adequadamente no domicílio.

Desse modo, em sua prática profissional, o enfermeiro não pode prescindir de intervenções direcionadas às necessidades das CCCs, visto que a primeira infância é um período crítico para o desenvolvimento físico, cognitivo e emocional. Nessa fase, ocorrem importantes formações cerebrais e aquisições que sustentam habilidades futuras mais complexas^(20)^. Portanto, é essencial garantir serviços de saúde especializados e atuação profissional qualificada, mesmo em contextos adversos, como a pandemia de COVID-19, assegurando que o cuidado familiar tenha caráter complementar, não substitutivo, ao atendimento profissional.

Este estudo evidenciou que a pandemia de COVID-19 foi compreendida pelas famílias como um agravante que ameaçava diretamente a vida das crianças com condição crônica pré-existente. Essa percepção reforçou a visão da criança (identidade da criança) como vulnerável e ampliou a consciência sobre a gravidade do seu quadro de saúde (visão da doença). Em resposta, as famílias incorporaram ou intensificaram medidas preventivas, como higiene rigorosa, uso de máscara e distanciamento social, práticas desafiadoras no cotidiano, mas mantidas como estratégia de manejo do cuidado (mentalidade de manejo). Tais adaptações revelam a capacidade de reorganização familiar diante das pressões impostas pela crise sanitária e evidenciam a necessidade de suporte profissional contínuo para sustentar a qualidade do cuidado.

É válido considerar que, durante a produção de dados em contexto pandêmico, ainda vigoravam medidas rigorosas de distanciamento social e persistiam incertezas quanto à doença, às suas consequências e à possibilidade de uma vacina. Esse cenário pode ter influenciado a visão das famílias sobre a criança e sua condição de saúde, visto que o contexto vivido tem potencial para modificar diretamente tais apreciações^(8)^. Embora a maioria das crianças infectadas pelo SARS-CoV-2 tenha apresentado sintomas leves^(21)^, os casos graves, hospitalizações e óbitos ocorreram predominantemente naquelas com comorbidades prévias^(21)^, justificando a preocupação dos cuidadores e a adoção ou intensificação de medidas preventivas para proteger suas CCCs. Além disso, as informações veiculadas pela mídia e pelas redes sociais durante a pandemia^(22)^ também podem ter contribuído para reforçar a percepção familiar sobre a vulnerabilidade das crianças e a gravidade de seu quadro de saúde.

Em ambos os contextos desta investigação, algumas famílias destacaram a perspectiva de normalidade em relação às suas crianças (identidade da criança). Embora não tenha sido a visão predominante, a normalização foi enfatizada por famílias cujas crianças apresentavam condições crônicas com demandas de cuidado menos complexas. Mesmo reconhecendo as fragilidades impostas pela condição crônica e a necessidade de cuidados contínuos, as famílias também expressaram sua percepção de normalidade. Esse achado sugere que as famílias estão na direção de um manejo efetivo do cuidado, podendo ser essa visão um recurso utilizado para cuidarem sem limitar a criança à presença de uma condição crônica.

Em relação à mentalidade de manejo, identificaram-se aspectos facilitadores do cuidado comuns aos dois contextos analisados, como a possibilidade de dedicação exclusiva à criança e o acesso ao plano de saúde. Essas percepções parecem refletir a perspectiva da família sobre a criança (identidade da criança), reconhecendo nela a necessidade de cuidado contínuo. A dedicação integral ao cuidado surge como estratégia para atender às múltiplas demandas, enquanto o acesso ao plano de saúde assume papéis distintos conforme o período: no contexto pré-pandemia, facilitava o atendimento domiciliar, reduzindo deslocamentos em meio à sobrecarga familiar; no contexto transpandemia, contribuiu para a manutenção dos atendimentos mesmo com suspensão dos serviços presenciais, demonstrando que, embora não seja uma facilidade produzida pelo contexto, sua função é diretamente influenciada por ele.

Revisão sistemática sobre a saúde de pais de CCCs indica que a dedicação exclusiva ao cuidado é um facilitador, devido à intensa rotina de cuidados domiciliares e consultas especializadas, muitas vezes incompatível com qualquer atividade remunerada do cuidador principal^(23)^. Contudo, essa centralização das demandas no cuidador principal pode aumentar sua sobrecarga. Este estudo evidenciou que os cuidadores eram predominantemente do sexo feminino e que a maioria das mães não trabalhava fora de casa, dedicando-se exclusivamente ao lar e à criança, em consonância com os achados da literatura, que apontam que as mulheres deixam de trabalhar para ficar em casa devido às demandas impostas pela condição crônica na infância^(24)^.

Sabe-se que, no decorrer da pandemia de COVID-19, as medidas de distanciamento social levaram a uma redução da rede de apoio das famílias de CCCs, fazendo com que a continuidade do cuidado das CCCs se tornasse, ainda mais, responsabilidade dos cuidadores principais^(25)^. Entretanto, conforme evidenciado neste estudo, a maior convivência familiar durante a pandemia favoreceu a maior coesão familiar, fortalecendo vínculos afetivos e a percepção de união para o cuidado. A mutualidade parental, portanto, contribui para um manejo mais estável e adaptativo, já que minimiza conflitos e distribui responsabilidades, reduzindo a sobrecarga individual e promovendo maior alinhamento nas ações^(26)^.

Observa-se que o contexto vivido pelas famílias de CCCs, especialmente durante a pandemia de COVID-19, impactou diretamente o manejo do cuidado, intensificando demandas, evidenciando vulnerabilidades e exigindo adaptação das rotinas familiares. Esses achados reforçam a necessidade de um olhar profissional atento e especializado, aliado ao desenvolvimento de políticas públicas e estratégias que apoiem efetivamente as famílias em situações de emergência.

São reconhecidas algumas limitações deste estudo. A produção de dados no contexto transpandemia ocorreu em um momento específico da pandemia de COVID-19, marcado por elevada incerteza, ausência de imunização e restrições rigorosas, o que pode ter influenciado as percepções familiares sobre a criança e o cuidado, limitando a extrapolação dos resultados para outros períodos da crise sanitária. Estudos envolvendo CCCs de maior complexidade, realizados em outros países ou em contextos socioculturais distintos, podem apresentar resultados diferentes, em função das variadas estratégias de enfrentamento da pandemia, das especificidades culturais e das políticas de saúde adotadas.

## CONCLUSÃO

Os achados deste estudo evidenciam que a perspectiva familiar sobre a CCC orienta o manejo do cuidado ao longo do tempo e é sensível às mudanças contextuais. No período pré-pandemia, uma perspectiva ancorada nas potencialidades da criança sustentou práticas voltadas à promoção do desenvolvimento infantil e à manutenção da saúde. No contexto transpandemia, a COVID-19 foi identificada como um agravante adicional à condição crônica, sendo percebida como ameaça direta à vida e contribuindo para a perspectiva familiar focada na fragilidade da criança. Isso resultou no aumento da vigilância e na incorporação de novas exigências de higiene na rotina familiar. Ainda assim, o reconhecimento de aspectos positivos da criança manteve-se como elemento mobilizador do cuidado, favorecendo a manutenção, no ambiente domiciliar, de atividades educacionais e de reabilitação pela família. Esses achados reforçam o caráter dinâmico da perspectiva familiar e sua centralidade no manejo do cuidado.

À luz desses achados, o estudo oferece implicações para a prática da enfermagem ao evidenciar a necessidade de que enfermeiros reconheçam a perspectiva familiar como elemento central na avaliação e no planejamento do cuidado da CCC, especialmente diante de mudanças em seus contextos de vida, como crises sanitárias. A utilização de referenciais teóricos, como o FMSF, pode auxiliar os profissionais a identificar como os cuidadores percebem a criança e organizam o cuidado no cotidiano, favorecendo intervenções centradas na família. No âmbito das políticas públicas, os resultados reforçam a importância da manutenção da continuidade do cuidado, do fortalecimento das redes de apoio e da organização de respostas assistenciais que considerem as demandas específicas das famílias em situações de crise, especialmente quando produzem mudança na oferta dos serviços demandados pelas crianças. Pesquisas futuras são necessárias para aprofundar a compreensão da perspectiva familiar em outros contextos e ao longo do tempo.

## DISPONIBILIDADE DE DADOS

Todo o conjunto de dados que dá suporte aos resultados deste estudo está disponível mediante solicitação ao autor correspondente.

# Translation: Perspectiva familiar sobre el cuidado de niños con condiciones crónicas en los contextos pre y transpandémicos de la COVID-19

**DOI:** 10.1590/1980-220X-REEUSP-2025-0448es

**Authors:** Nayara Luiza Henriques, Maísa Mara Lopes Macêdo Breves, Melissa Joice de Abreu Felizardo, Gabriel Lucas Santos Galdino, Samylla Alves de Almeida, Elysângela Dittz Duarte

**Affiliations:** 1 Universidade Federal de Minas Gerais, Escola de Enfermagem, Departamento de Enfermagem Materno-Infantil e Saúde Pública, Belo Horizonte, MG, Brasil.

**Keywords:** Enfermedad Crónica, Cuidado del Niño, Familia, COVID-19, Atención de Enfermería

## Abstract

**Objetivo:**

Comprender las perspectivas de las familias sobre el cuidado de niños con condiciones crónicas en los contextos pre y transpandémicos de la COVID-19, a la luz del Family Management Style Framework.

**Métodos:**

Estudio cualitativo longitudinal con 24 cuidadores familiares de niños de 2 a 4 años. Los datos se obtuvieron mediante un cuestionario socioeconómico y entrevistas semiestructuradas en dos etapas: prepandemia (octubre/2019 a mayo/2020) y transpandemia (junio/2020 a enero/2021). Posteriormente, se sometieron a un análisis temático guiado por el marco teórico.

**Resultados:**

La perspectiva familiar orientó la gestión de los cuidados a lo largo del tiempo y se vio modificada por la pandemia. En el contexto prepandémico, el enfoque se centraba en el potencial del niño, orientando las acciones hacia el desarrollo y el mantenimiento de la salud. En el contexto transpandémico, la perspectiva se centró en las debilidades y vulnerabilidades del niño, asociadas a la percepción de amenaza por la COVID-19, lo que resultó en una mayor vigilancia y atención higiénica.

**Conclusión:**

La perspectiva familiar orienta el manejo del cuidado y es sensible a los cambios contextuales, ofreciendo subsidios para la cualificación de la práctica profesional en contextos de crisis sanitaria.

## INTRODUCCIÓN

Incorporación de nuevas tecnologías en salud ha contribuido a la creciente especialización de la atención materno-infantil^(1)^, lo que se ha reflejado en la reducción de las tasas de mortalidad infantil y en el aumento del número de niños que viven con alguna condición crónica^(2)^. Las condiciones crónicas en la infancia son aquellas de origen congénito o adquirido, con base biológica, psicológica o cognitiva, que dan lugar a limitaciones funcionales permanentes o temporales^(3)^.

En el ámbito doméstico, las familias son las protagonistas del cuidado de los niños con condiciones crónicas (NCCs)^(4)^. Las familias conviven con el desafío constante de adaptar a la rutina la ejecución de los cuidados esenciales para el mantenimiento de la salud de los niños, que incluyen desde la alimentación y la higiene hasta el manejo de dispositivos tecnológicos complejos^(5)^. Además de las tareas domésticas, los cuidadores a menudo necesitan movilizar recursos para asistir regularmente a consultas especializadas y reorganizar la vida familiar en caso de hospitalizaciones^(6)^. Este conjunto de responsabilidades, inherentes al curso de vida de estos niños, construye una vida cotidiana caracterizada por la sobrecarga y el estrés familiar^(7)^.

La magnitud de esta cuestión se ve aún más acentuada por el contexto en el que se inserta la familia. Según el Marco de referencia del estilo de gestión familiar (*Family Management Style Framework* - FMSF)^(8)^, modelo teórico que ayuda a comprender el manejo familiar de la condición crónica en la infancia, el contexto constituye una dimensión central del cuidado familiar, capaz de modificar las condiciones de vida de la familia y la manera en que esta percibe y gestiona el cuidado^(8)^.

Se experimentó un cambio contextual repentino a nivel mundial con la llegada de la pandemia de *Coronavirus Disease 2019* (COVID-19) en marzo de 2020^(9)^. Debido al elevado potencial de propagación del virus, los decretos gubernamentales establecieron medidas de distanciamiento social, que dieron lugar a la suspensión de la atención médica electiva en salud y al cierre de servicios, como escuelas y guarderías, además de la adopción de medidas de protección, como el uso de cubrebocas y la intensificación de la higiene de las manos^(9)^. Las implicaciones de este cambio contextual afectaron la vida de todas las personas, especialmente de las familias de niños con condiciones crónicas. Además de realizar los cuidados de rutina que requieren los niños, incorporaron nuevas medidas de higiene y vivieron la restricción de la convivencia social, así como la suspensión de los seguimientos en salud y de las actividades escolares. De este modo, el contexto de crisis sanitaria impuso exigencias adicionales a las familias, que ya tenían una sobrecarga de tareas, al mismo tiempo que redujo sus redes de apoyo^(10)^.

Ante los cambios sustanciales en el cuidado de los niños con condiciones crónicas provocados por la pandemia de COVID-19, sobre todo en comparación con el momento anterior a la pandemia, se parte del supuesto de que la familia modificó su perspectiva sobre el niño y su estado de salud, lo que, a su vez, repercutió directamente en la gestión familiar del cuidado. Esto se debe a que las acciones de cuidado dirigidas a los niños con condiciones crónicas, que son las respuestas activas de la familia ante sus necesidades, dependen de cómo son reconocidas y vistas por sus familias^(8)^. Así, la perspectiva familiar sobre las capacidades y necesidades de los niños con condiciones crónicas moldea directamente la manera en que se lleva a cabo el cuidado^(8)^.

Se observa un número creciente de estudios que se centran en la sobrecarga y el impacto emocional que puede generar el cuidado de los NCC. Sin embargo, la literatura disponible se ha dedicado predominantemente a estos aspectos, prestando menor énfasis al análisis de la perspectiva de las familias sobre el cuidado en contextos distintos y marcados por cambios abruptos, como la pandemia de COVID-19.

Además, aunque los estudios desarrollados durante la pandemia abordaron las transformaciones ocurridas en el cuidado familiar, se basaron, en su mayoría, en datos recopilados tras el inicio de la crisis sanitaria, lo que limita el análisis de la perspectiva familiar a lo largo del tiempo y ante el cambio contextual, un vacío de conocimiento que aborda esta investigación.

Ante este escenario, se definió la siguiente pregunta de investigación: ¿cuál es la perspectiva de las familias sobre el cuidado de un NCC en los contextos pre y transpandémicos de la COVID-19? Para responder a esta pregunta, el estudio tiene como objetivo comprender la perspectiva de las familias sobre el cuidado de los NCC en los contextos pre y transpandémicos de la COVID-19 a la luz del FMSF.

## MÉTODO

### Diseño del Estudio

Se trata de un estudio cualitativo longitudinal^(11)^, cuya elaboración siguió las recomendaciones de transparencia descritas en la herramienta de consenso denominada Criterios Consolidados de Informe de Investigación Cualitativa (COREQ), versión brasileña^(12)^.

### Marco Teórico-Metodológico

Para esta investigación, se adoptó como marco teórico el Marco de referencia del estilo de gestión familiar (Family Management Style Framework - FMSF)^(8)^, modelo desarrollado por las enfermeras Kathleen Knafl y Janet Deatrick para comprender cómo las familias perciben y responden activamente a las demandas de cuidado de un niño con necesidades especiales, considerando el contexto en el que se insertan. Como marco metodológico, se adoptó el análisis temático propuesto por Braun y Clarke^(13)^, que permite la interpretación de los datos de la investigación a partir de categorías previamente definidas y fundamentadas en el marco teórico. En este estudio, el análisis se centró en uno de los componentes del FMSF, denominado “definición de la situación”, que permite comprender la perspectiva de la familia sobre el cuidado del NCC.

### Participantes

Participaron 24 cuidadores familiares de NCC de entre 2 y 4 años de edad, altas de dos Unidades de Terapia Intensiva Neonatal (UTIN) vinculadas a un hospital filantrópico y a un hospital federal, ambos ubicados en una capital de la región sudeste de Brasil. Las instituciones fueron elegidas por ser referentes nacionales del Sistema Único de Salud en el cuidado materno-infantil.

### Definición de la Muestra y Criterios de Selección

La definición de la muestra fue intencional y no probabilística, basada en criterios de elegibilidad. Se incluyeron cuidadores familiares responsables del cuidado de NCC de entre 2 y 4 años de edad, considerando que las alteraciones en el desarrollo neuropsicomotor se vuelven más evidentes en este rango de edad^(14)^. Los cuidadores debían tener 18 años o más, ser localizables por teléfono y residir en el mismo domicilio que el niño, ya que se les consideró informantes clave de la dinámica familiar involucrada en garantizar el cuidado del niño con condiciones crónicas en el entorno doméstico; por lo tanto, era fundamental que compartieran el mismo espacio de convivencia^(15)^. Se excluyó a los cuidadores con dificultades de comunicación o alteraciones psicológicas y/o psiquiátricas que imposibilitaran la recolección de datos, así como a aquellos que no respondieron tras tres intentos consecutivos de contacto telefónico.

### Recolección de Datos

El estudio se llevó a cabo en dos etapas, correspondientes a los dos contextos investigados: etapa 1 (contexto prepandémico de la COVID-19), que abarcó el período de octubre de 2019 a mayo de 2020, durante la cual se llevó a cabo la primera fase de recopilación de datos con las familias, antes de que la Organización Mundial de la Salud declarara la COVID-19 como pandemia el 11 de marzo de 2020^(9)^; y la etapa 2 (contexto durante la pandemia o transpandemia), referente al período comprendido entre junio de 2020 y enero de 2021, considerado en este estudio como el intervalo en el que se produjo la recopilación de datos en el contexto de la pandemia, caracterizando el transcurso de esta situación sin precedentes en el mundo^(16)^.

La identificación de los participantes se llevó a cabo en la primera etapa del estudio (contexto prepandémico). Para ello, se analizaron todos los expedientes médicos de los niños que recibieron el alta de la UTIN entre diciembre de 2016 y diciembre de 2017, con el objetivo de incluir a niños de entre 2 y 4 años.

A partir del análisis de los expedientes médicos, se identificaron 1.115 cuidadores, de los cuales 852 procedían del hospital filantrópico y 263 del hospital federal. Entre octubre de 2019 y mayo de 2020, se contactó a todos por teléfono para aplicarles el Cuestionario para la Identificación de Niños con Condiciones Crónicas – Revisado (QuICCC-R), versión brasileña, instrumento utilizado para detectar los NCC^(17)^. Del total de cuidadores identificados, no fue posible establecer contacto con 829, debido a números inexistentes o desactualizados. Así, se contactó a 286 cuidadores. De ellos, diez se negaron a participar; cinco informaron que sus niños habían fallecido; y 218 tenían niños que no presentaban condiciones crónicas según el QuICCC-R, lo que dio como resultado 53 cuidadores participantes en la Recolección de datos de la primera etapa (prepandémica).

Con la llegada de la pandemia de COVID-19, el estudio se amplió, con el consentimiento del Comité de Ética, incluyendo una segunda etapa que se refería al contexto transpandémico. Se intentó contactar a los mismos 53 cuidadores que participaron en la primera etapa. De ellos, nueve ya no tenían el mismo número de teléfono; 13 no respondieron al contacto después de tres intentos; y cuatro no aceptaron participar. Quedaron, así, 27 cuidadores, de los cuales tres formaron parte de un estudio piloto para la validación del guion de las entrevistas y no se incluyeron en el análisis de datos. De este modo, la población definida para este estudio estuvo compuesta por participantes que estuvieron presentes en ambas etapas, lo que totalizó 24 cuidadores. Cabe destacar, además, que, en los dos momentos de la Recolección de datos, se consideró como encuestado al mismo familiar.

En la primera etapa del estudio, tras la confirmación de la condición crónica del niño, se invitó a las familias a participar en el estudio y se programaron las entrevistas. Inicialmente, estas se realizaron en el domicilio de las familias.

Sin embargo, debido a la dificultad de desplazamiento de las investigadoras al interior de Minas Gerais, donde residía la mayoría de las familias, y con la aprobación del Comité de Ética, las entrevistas pasaron a realizarse por teléfono. En la segunda etapa, relativa al contexto transpandémico, y teniendo en cuenta las normas de seguridad implementadas durante la pandemia, todas las entrevistas se realizaron por teléfono y se grabaron tras el consentimiento verbal del participante.

En ambas etapas, se aplicó un cuestionario socioeconómico para realizar la recolección de información sobre el niño (sexo, edad gestacional y cronológica y diagnóstico) y el familiar (grado de parentesco, edad, etnia, nivel educativo, situación conyugal, ocupación e ingresos). A continuación, se utilizó un guion semiestructurado, elaborado a partir del FSMF^(8)^. En ambas fases, el enfoque de las entrevistas fue la perspectiva familiar sobre el cuidado de los NCC. Las entrevistas fueron realizadas por dos investigadoras, maestras y doctorandas en enfermería, con experiencia en entrevistas cualitativas y sin vínculo previo con las familias.

### Análisis y Tratamiento de Datos

Las entrevistas se transcribieron íntegramente y se sometieron a un análisis temático de tipo deductivo guiado por el marco teórico del FMSF. Para ello, se adoptó el proceso analítico propuesto por Braun y Clarke^(13)^, compuesto por seis fases: 1) Familiarización con los datos mediante la transcripción y lectura de las entrevistas; 2) Generación inicial de códigos preliminares basados en el marco teórico, abarcando el componente “definición de la situación” del FMSF y sus respectivas dimensiones conceptuales: identidad del niño; visión de la condición; mentalidad de manejo; y mutualidad entre los padres; 3) Elaboración de resúmenes de los fragmentos codificados de las entrevistas y reconocimiento de patrones; 4) Revisión de los patrones identificados para garantizar su distinción y consistencia; 5) Elaboración de definiciones para cada patrón/tema identificado; 6) Descripción y análisis de los resultados, relacionándolos con el marco teórico y el objetivo del estudio.

### Aspectos Éticos

La investigación se llevó a cabo de conformidad con las Resoluciones n.º 466/2012 y n.º 580/2018 del Consejo Nacional de Salud^(18,19)^, y fue aprobada por el Comité de Ética en Investigación mediante el Dictamen n.º 3.508. 414/2019 y la Enmienda n.º 4.331.516/2020. Los participantes firmaron el Formulario de Consentimiento Libre e Informado en dos ejemplares. Con el fin de garantizar la confidencialidad y el anonimato, sus nombres fueron sustituidos en las entrevistas por códigos alfanuméricos utilizando las letras M (“madre”), P (“padre”), T (“tía”) y C (“niño”). Las letras fueron seguidas por el número correspondiente al orden de realización de las entrevistas (p. ej.: M4, P3, T1).

## RESULTADOS

### Caracterización de las Familias y los Niños con Condiciones Crónicas

La mayoría de los cuidadores participantes eran madres (n = 22). Solo se realizó una entrevista con el padre y otra con la tía del niño con condición crónica.

La edad media de los encuestados fue de 34 años, siendo la mínima de 19 y la máxima de 54 años. La mayoría de los participantes se declararon casados (n = 12), de raza mestiza (n = 15), con estudios superiores completos (n = 8), residiendo con su pareja (n = 20) en el interior de Minas Gerais (n = 16). Los ingresos familiares declarados oscilaron entre uno y dos salarios mínimos (n = 11), lo que, en ese momento, correspondía a un valor de R$1.100,00. Siete cuidadores declararon ingresos inferiores a un salario mínimo. De las madres participantes, 14 no formaban parte del mercado laboral y se dedicaban al cuidado del NCC. En el contexto de la pandemia, 17 participantes informaron haber recibido algún tipo de ayuda económica. En cuanto a los niños con condiciones crónicas, la mayoría eran varones (n = 15), con una edad media de 2 años y 8 meses. La edad gestacional media al nacer fue de 31,8 semanas. Los diagnósticos médicos predominantes fueron de naturaleza neurológica (n = 10).

### Perspectiva de las Familias Sobre el Cuidado de los Niños con Enfermedades Crónicas

El análisis de datos se orientó a comprender la perspectiva de las familias sobre el cuidado de los niños con enfermedades crónicas. Para ello, se utilizó el componente conceptual “definición de la situación” del FMSF, que permite comprender cómo los cuidadores perciben al niño y su enfermedad crónica, así como la influencia de esa percepción en la gestión del cuidado. Este componente abarca cuatro dimensiones exploradas en este estudio: identidad del niño, visión de la enfermedad, mentalidad de manejo y mutualidad parental.

Se realizó una comparación de la información generada en los contextos pre y transpandémicos de la COVID-19 para identificar patrones y variaciones en las perspectivas de las familias a lo largo del tiempo, evidenciando situaciones de mejora, empeoramiento o ausencia de modificaciones. Esta síntesis se representa en la Figura 1, en la que se destaca cada dimensión del componente “definición de la situación”, y las líneas punteadas indican la interconexión entre ellas según el discurso de los participantes. A continuación de la figura, se presentará cada una de las dimensiones en detalle.

**Figura 1:** Perspectiva de la familia sobre el cuidado del niño con una condición crónica en los contextos prepandémico y transpandémico de la COVID-19 – Belo Horizonte, MG, Brasil, 2025.

### Identidad del Niño

Según el FMSF, la identidad del niño se refiere a la perspectiva de la familia sobre el NCC, teniendo en cuenta el reconocimiento de sus potencialidades y vulnerabilidades^(8)^. En este estudio, en el contexto prepandémico, la mayoría de las familias enfatizó las potencialidades de los niños, como habilidades adquiridas (ponerse de pie, hablar, correr, alimentarse por sí mismos) y características positivas (independencia, inteligencia, buena memoria y facilidad para aprender). En cambio, en el contexto de la pandemia, la perspectiva cambió y se centró en las fragilidades, relacionándolas especialmente con la baja inmunidad, el compromiso pulmonar o el reconocimiento de que los niños pequeños son susceptibles de enfermarse de COVID-19. De este modo, los patrones de empeoramiento en la perspectiva fueron los más recurrentes ante el cambio contextual.

La ausencia de cambios en la perspectiva familiar se evidenció en el reconocimiento de que los niños requieren cuidados continuos y vigilancia constante en ambos contextos estudiados. Es decir, el enfoque se mantuvo en las vulnerabilidades del niño antes y durante la pandemia de COVID-19. Esta percepción fue más frecuente entre las familias de niños con condiciones de salud que exigían cuidados más complejos. Por otro lado, entre aquellas cuyas niñas y niños presentaban necesidades de cuidado menos complejas, la perspectiva se mantuvo centrada en la normalidad de la niña o el niño a lo largo de los dos momentos investigados.

Se identificó solo una situación de mejoría, relatada por M1, cuyo niño presentaba, en el contexto prepandémico, dificultades para caminar debido a un pie zambo congénito. Con la resolución del problema a lo largo del tiempo, hubo un cambio positivo en la percepción de la familia relacionada con la mejoría del cuadro de salud del niño, no con el contexto pandémico. El Cuadro 1 presenta la información relativa a la perspectiva de las familias sobre el NCC. En la primera columna se encuentran las perspectivas familiares acompañadas de la mención de las familias que las relataron; en la segunda, se encuentra el patrón de variación observado (mejora, empeoramiento o ausencia de modificación); y en la tercera, se encuentran ejemplos de enunciados que ilustran la perspectiva a lo largo del tiempo.

**Cuadro 1:** Identidad del niño – Belo Horizonte, MG, Brasil, 2025.

Identidad del niño			
Perspectiva de la familia	Patrón de variación de la perspectiva	Datos empíricos	
		Contexto prepandémico	Contexto transpandémico
Susceptibilidad del niño a enfermar en el contexto de la transpandemia^(M1, M2, M3, M5, M6, M8, M9, M14, M16, M17, M18, M21 y M22)^	Empeoramiento	*Es un niño tranquilo, no es de esos niños que viven en el hospital, no. En cuanto a su salud, está tranquilo [...] ¡y ya sabe hablar y se mantiene de pie! (M1)*	*Lo que realmente me da miedo es que se contagie del coronavirus. Como tiene bronquitis, creo que es más fácil que se contagie. Su sistema inmunitario está más debilitado, ¿no? Pertenece a un grupo de riesgo. (M1)*
Necesidad de cuidados continuos en ambos contextos^(M8, M12 y M13)^	Sin modificaciones	*C12 necesita atención las 24 horas. Para todo lo que hace, depende de otra persona. (M12)*	*Es una niña que necesita cuidados todo el tiempo, para todo: para comer, para bañarse, para desplazarse. Su motricidad está totalmente comprometida. (M12)*
Visión de la normalidad en ambos contextos^M3, P4, M6, M7, T11, P15, M20, M23, M24^	Sin modificaciones	*Ya vemos a C15 como una niña normal, porque es muy inteligente. Su desarrollo es bueno. (P15)*	*Su desarrollo es excelente. Durante la pandemia, gracias a Dios, se está desarrollando bien. Todo normal. (P15)*
Capacidad para realizar actividades ^(M2, M3, M4, M5, M6, M7, M9, M10, T11, M12, M13 y M14)^	Sin cambios	*Gracias a Dios, ha mejorado mucho. Hoy en día, ya se mantiene de pie sola; da unos pasitos; vemos su progreso. (M6)*	*En cuanto al habla, ya he conseguido algunos avances. Ya dice “mamá”, “papá”, “aquí”, “¡eh!”, algunas cositas monosilábicas. (M6)*
Capacidad para realizar actividades^(M1)^	Mejora	*El C1 se cae constantemente y me da miedo que se caiga, porque se golpea la cara contra el suelo y me da miedo que se golpee la cabeza. (M1)*	*Ya no se cae tanto como antes. Tenía los piececitos un poco hacia dentro, pero se lo arreglaron y dejó de caerse. (M1)*

### Percepción de la Enfermedad

Según la FMSF, la percepción de la enfermedad se refiere a la comprensión que tiene la familia del estado de salud del niño^(8)^. En este estudio se observó que, en el periodo prepandémico, las familias eran conscientes de la gravedad del estado de salud de los niños con CC. Con la llegada de la pandemia, empezaron a percibir que la infección por el virus SARS-CoV-2 representaba un riesgo adicional, lo que constituía una amenaza directa para la vida de sus hijos y agravaba la perspectiva de gravedad de su estado de salud. Así, solo se identificaron situaciones de empeoramiento en la percepción de la enfermedad, tal y como se ejemplifica en el Cuadro 2.

**Cuadro 2:** Percepción de la enfermedad – Belo Horizonte, MG, Brasil, 2025.

Percepción de la enfermedad			
Perspectiva de la familia	Patrón de variación de la perspectiva	Datos empíricos	
		Contexto prepandémico	Contexto transpandémico
Percepción de la gravedad del cuadro clínico del niño asociada al riesgo potencial de empeoramiento por COVID-19^(M1, M2, M3, M5, M6, M8, M9, M14, M16, M17, M18, M21, M22 y M24)^	Empeora	*El C24 tiene una cardiopatía congénita grave, ¿no? Entonces, hacemos los seguimientos como es debido. (M24)*	*Si contrae el virus, su cardiopatía podría empeorar aún* más; *no sé si se salvará. (M24)*

### Mentalidad de Manejo

Según la FMSF, la mentalidad de manejo abarca la perspectiva de la familia sobre las facilidades y/o dificultades a la hora de llevar a cabo el régimen de cuidados del NCC^(8)^. Al comparar los discursos de las familias en ambos contextos investigados, se observa que los patrones de empeoramiento en sus perspectivas son los más frecuentes. Estos se caracterizan por las dificultades mencionadas en en relación con el contexto de la pandemia de COVID-19, como la intensificación de las medidas de higiene para contener la enfermedad y la restricción de la familia al domicilio, lo que provocó repercusiones emocionales como ansiedad y estrés. La interrupción de las terapias de rehabilitación y educación durante la pandemia culminó en la necesidad de que la familia asumiera y realizara estas actividades en el domicilio, situaciones que condujeron a una peor perspectiva sobre la gestión de los cuidados. M13 relata una dificultad que mantuvo en ambos contextos del estudio relacionada con las múltiples consultas a las que acudía su hijo. En el contexto prepandémico, el reto de la madre era organizarse para estar presente en las citas. En el contexto de la pandemia, la dificultad estuvo relacionada con la interrupción de las citas.

En cuanto a los factores que facilitaron el cuidado, que se mantuvieron sin cambios a lo largo del tiempo, destacaron la continuidad de las terapias y la atención sanitaria incluso durante la pandemia, la posibilidad de dedicarse en exclusiva a los hijos y la existencia de un seguro médico. Solo se informó de una mejora por parte de M1, relacionada con la reducción del número de consultas debido a la evolución clínica del niño, lo que facilitó la gestión del cuidado. En este caso, la mejora reflejó el estado de salud del niño, no el contexto pandémico. El Cuadro 3 ilustra la información presentada.

**Cuadro 3:** Mentalidad de manejo – Belo Horizonte, MG, Brasil, 2025.

Mentalidad de manejo			
Perspectiva de la familia	Patrón de variación de la perspectiva	Datos empíricos	
		Contexto de la pandemia de COVID-19	
Dificultades relacionadas con la interrupción de los servicios de salud y educación^(M2, M5, M6, M7, M10, T11, M16 y M22)^	Empeora	*La cosa está así: un ajetreo, ¿no? Más estresante, porque no hay colegio, ¿no? Entonces, se quedan en casa más ansiosos. (M7)* *Tuvimos que dejar las clases de natación. La consulta del hospital no atendía. Aquí en la APAE, el médico también dejó de atender, así que la atención se volvió difícil. (M22)*	
Dificultad debido al mantenimiento de las actividades de rehabilitación y educación en el domicilio^(M2, M5, M6, M7, M10, T11, M16 y M22)^	Empeoramiento	*No se están realizando esas sesiones presenciales. Entonces, en ese caso, se está haciendo fisioterapia online, ¿no? Me llaman, hacen una videollamada y, en ese caso, soy yo quien tiene que hacer fisioterapia. (M10)* *Me mandan las actividades del colegio para que se las enseñe. No es fácil, ¿sabes? (C6)*	
Dificultad debido al aumento de las medidas de higiene^(M1, M3, M8, M9, M23 y M24)^	Empeora	*Estoy siendo más cautelosa con C1. No salgo tanto como antes, siempre le estoy lavando las manitas; cuando salgo, llego a casa y enseguida le cambio la ropa, le doy un baño. Mi cuidado con él se ha duplicado. (M1)* *Limpio todo a diario con lejía para desinfectarlo. A las cosas de C8 les echo alcohol. Ahora, con la pandemia, es muy difícil mantener sus cuidados y estos cuidados, pero los sigo haciendo todos. (M8)*	
Dificultad debido a la restricción de la niña y la familia en el domicilio^(M1, M3, M7, M10, M13, M14, M17 y M19)^	Empeora	*Estar más encerrado en casa es malo, ¿no? Acabamos estresándonos más. No se puede salir bien, no hay libertad. (M1)* *Ah, ha habido un cambio, ¿no? Porque nos hemos quedado todos en casa, sobre todo para mí. Para mí, es muy agotador y estresante. (M13)*	
Facilidad para mantener los tratamientos durante la pandemia^(M1, M2, M3, M6, M7, M9, T11)^	Sin cambios	*Gracias a Dios, la equinoterapia sigue funcionando. Es la única actividad autorizada en mi ciudad. (M3)* *Les hacen un seguimiento en la APAE, y está siendo muy importante para ellos ahora durante la pandemia. (M7)*	
Facilidad debido al acceso del niño al seguro médico^(M6 y T11)^	Sin modificaciones	*La C6 tiene un acuerdo desde hace ya dos años. El médico viene a casa, la fisioterapia también, así que no hace falta salir con ella. (M6)* *A pesar de la pandemia, siguió prestando asistencia. Eso fue estupendo. Tener un plan marca la diferencia en momentos como estos, ¿no? (T11)*	
Facilidad gracias a la posibilidad de dedicarse al cuidado ^(M1, M8)^	Sin modificaciones	**Contexto prepandémico**	**Contexto transpandémico**
		*Sí, tranquilo. Como no trabajo, me quedo en casa por el C1. (M1)*	*Me quedo a cargo de él, lo que me facilita mucho las cosas, así que hago todo lo que puedo por él. (M1)*
Dificultad en relación con las múltiples citas médicas^(M13)^	Sin cambios	*Tengo que organizar mis horarios en función de las terapias de C13. Es muy duro para mí. (M13)*	*El único problema es conseguir atención médica para ella en plena pandemia. Sin eso, su desarrollo se retrasa por completo. (M13)*
Dificultad en relación con las múltiples citas médicas^(M1)^	Mejora	*Son muchos médicos, ¿no?, muchas cositas, y yo, que soy madre primeriza, lo veo más complicado [...] va a ver a muchos profesionales. (M1)*	*Siempre lo estoy llevando al médico. Lo bueno es que no se puede comparar con antes. Lo llevo más a las consultas de rutina y cuando le da la bronquitis. (M1)*

### Reciprocidad de Responsabilidades Entre los Padres

Según el FMSF, la reciprocidad parental se refiere a las creencias de la familia sobre el grado en que comparten o difieren sus perspectivas respecto al cuidado de sus hijos^(8)^. En este estudio, no se observó un empeoramiento en esta dimensión. La mayoría de las familias mostraron una alineación entre sus miembros en la toma de decisiones y en el cuidado de los niños. Incluso ante el cambio de contexto, los cuidadores que residían con sus parejas mantuvieron un diálogo constante y decisiones compartidas, equilibrando las necesidades del niño.

Algunos relatos destacaron que mantener una visión de normalidad y el sentimiento de amor constituían un factor que reforzaba el cuidado. Por el contrario, otros pusieron de manifiesto la dificultad para aceptar el estado de salud del niño o la falta de apoyo por parte de la pareja en ambos contextos del estudio. Se identificó una mejora en la perspectiva familiar en los discursos de P4 y M7, quienes afirmaron que la pandemia de COVID-19 aumentó la convivencia y la unión familiar debido al mayor tiempo de permanencia juntos en el hogar. El Cuadro 4 presenta la síntesis de estas perspectivas.

**Cuadro 4:** Reciprocidad parental – Belo Horizonte, MG, Brasil, 2025.

Mutualidad parental			
Perspectiva de la familia	Patrón de variación de la perspectiva	Datos empíricos	
		Contexto prepandémico	Contexto transpandémico
Unión y reparto de las tareas de cuidado^(M2, M3, P4, M6, M7, M10, M13, P15, C20, C21)^	Sin modificaciones	*Lo que más tengo en común con mi marido es el cuidado que ponemos en el C3. En este aspecto, estamos muy unidos y nos parecemos mucho. (M3)*	*Gracias a Dios, no tenemos ninguna discrepancia. Estamos muy unidos para cuidar del C3. (M3)*
Diálogo y decisiones compartidas^(M3, M7, P15, M16 y M24)^	Sin modificaciones	*Mantenemos un diálogo sobre todo lo que tiene que ver con C16 y discutimos todas las decisiones. (M16)*	*Mi marido y yo hablamos mucho, tomamos las decisiones juntos, incluso sobre eso de no recibir más visitas durante la pandemia. (M16)*
Ausencia de diálogo y perspectivas distintas sobre el cuidado^(M14)^	Sin modificaciones	*Mi marido no me ayuda. Tampoco hablamos de eso, así que al final lo dejo pasar. Ya tenemos demasiados problemas. (M14)*	*A mi marido no le preocupa. Ni siquiera puedo pedirle ayuda. No me atrevo a hablar de esto con él, así que resuelvo las cosas yo sola. (M14)*
Perspectiva de normalidad sobre el niño^(M3 y M23)^	Sin modificaciones	*Mi marido y yo pensamos igual. Tenemos que llevar la vida de C23 como la de cualquier otro niño normal. (M23)*	*La tratamos igual que a nuestro otro hijo. Para nosotros, es una niña normal. (M23)*
No aceptación del estado de salud del niño^(M8)^	Sin cambios	*Mi marido no lo acepta en absoluto. Yo tampoco lo acepto, pero nos conformamos con que fue Dios quien lo permitió. (M8)*	*Mi marido también se ha sentido un poco agobiado, igual que yo, porque es algo difícil de aceptar... no lo aceptamos. (M8)*
Perspectiva similar sobre la educación del niño^(M7, P15 y M18)^	Sin modificaciones	*Mi visión respecto a su educación para el futuro es la misma que la de mi marido. (M18)*	*Valoramos mucho su educación, le enseñamos a ser educado todos los días. (M18)*
Amor^(M8 y M10)^	Sin modificaciones	*Su padre y yo compartimos un gran cariño por ella. La queremos muchísimo. (M10)*	*Le tenemos mucho cariño y, por eso, la cuidamos muy bien juntos. (M10)*
Convivencia y estrechamiento de los lazos familiares ^(P4 y M7)^	Mejora	**Contexto transpandémico**	
		*Hemos tenido más diálogo y compañerismo en casa. La pandemia nos ha unido mucho gracias a la mayor convivencia. (P4)* *La pandemia nos ha brindado la oportunidad de estar más unidos, juntos, hablar más sobre las cosas y cuidar de C7. (M7)*	

## DISCUSIÓN

El análisis de los datos permitió comprender la perspectiva de las familias sobre sus hijos y su estado de salud en el contexto previo y durante la pandemia de COVID-19. En resumen, el conjunto de la información obtenida permite reconocer que la pandemia, como cambio contextual, tuvo implicaciones en la perspectiva de las familias de niños con CC.

Según el FMSF, la visión que la familia tiene del niño y su estado de salud influye significativamente en la gestión de los cuidados^(8)^, ya que las acciones de cuidado dirigidas a los niños con enfermedades crónicas dependen de cómo los ven sus familias. De este modo, cada una de las dimensiones de la perspectiva de las familias (identidad del niño, visión de la enfermedad, mentalidad de manejo y mutualidad parental) está íntimamente ligada y constituye la base familiar para el cuidado familiar del niño con enfermedades crónicas^(8)^.

Las interconexiones entre las dimensiones de la perspectiva familiar, presentadas en la Figura 1, evidencian cómo esta percepción orientó el manejo de la atención a lo largo del tiempo. En el contexto prepandémico, la perspectiva familiar enfocada en las potencialidades de los niños con condiciones crónicas (identidad del niño) justificó las acciones de cuidado orientadas al mantenimiento de la salud y a la promoción del desarrollo infantil (mentalidad de manejo). Al reconocer los avances en el desarrollo de sus hijos, así como las habilidades que estos son capaces de adquirir, las familias dirigieron sus esfuerzos a garantizar que dichos avances fueran continuos. Ya en el contexto transpandémico, aunque la visión predominante de la familia no se centraba en las potencialidades de los NCC, el reconocimiento de los aspectos positivos sustentó el esfuerzo familiar por mantener, en el hogar, las actividades de enseñanza y rehabilitación interrumpidas por los servicios de salud y educación.

Es importante destacar que, en el contexto de la transpandemia, las familias reconocieron como una dificultad la necesidad de mantener las actividades de rehabilitación y educación en el hogar (mentalidad de manejo). Sin embargo, fue una respuesta activa a la suspensión de las consultas electivas y las actividades escolares, lo que refleja su compromiso por asegurar la continuidad del estímulo al desarrollo de sus hijos. Esta información destaca la importancia de la competencia familiar para el mantenimiento de la atención en situaciones de fragilidad en la oferta de servicios de salud y reafirma la relevancia de la participación del personal de enfermería en el desarrollo constante de estas competencias, de modo que las familias, al ser una constante en la vida de los niños, logren actuar para garantizar la continuidad de la atención, incluso en contextos inesperados. Sin embargo, se debe considerar un límite a la capacidad de las familias para mantener estas actividades, ya que las necesidades de los niños pequeños cambian con el paso del tiempo y es posible que ya no puedan atenderse adecuadamente en el hogar.

De este modo, en su práctica profesional, el enfermero no puede prescindir de intervenciones dirigidas a las necesidades de los niños pequeños, ya que la primera infancia es un período crítico para el desarrollo físico, cognitivo y emocional. En esta fase, se producen importantes formaciones cerebrales y adquisiciones que sustentan habilidades futuras más complejas^(20)^.

Por lo tanto, es esencial garantizar servicios de salud especializados y una actuación profesional calificada, incluso en contextos adversos, como la pandemia de COVID-19, asegurando que el cuidado familiar tenga un carácter complementario, y no sustitutivo, de la atención profesional.

Este estudio puso de manifiesto que las familias percibieron la pandemia de COVID-19 como un agravante que amenazaba directamente la vida de los niños con una condición crónica preexistente. Esta percepción reforzó la visión del niño (identidad del niño) como vulnerable y amplió la conciencia sobre la gravedad de su estado de salud (visión de la enfermedad). En respuesta, las familias incorporaron o intensificaron medidas preventivas, como la higiene rigurosa, el uso de cubrebocas y el distanciamiento social, prácticas desafiantes en la vida cotidiana, pero mantenidas como estrategia de manejo del cuidado (mentalidad de manejo). Tales adaptaciones revelan la capacidad de reorganización familiar ante las presiones impuestas por la crisis sanitaria y evidencian la necesidad de apoyo profesional continuo para sostener la calidad del cuidado.

Es válido considerar que, durante la recopilación de datos en el contexto de la pandemia, aún estaban vigentes medidas rigurosas de distanciamiento social y persistían incertidumbres respecto a la enfermedad, sus consecuencias y la posibilidad de una vacuna. Este escenario puede haber influido en la visión de las familias sobre el niño y su estado de salud, dado que el contexto vivido tiene el potencial de modificar directamente tales apreciaciones^(8)^. Aunque la mayoría de los niños infectados por el SARS-CoV-2 presentaron síntomas leves^(21)^, los casos graves, las hospitalizaciones y las muertes se produjeron predominantemente en aquellos con comorbilidades previas^(21)^, lo que justifica la preocupación de los cuidadores y la adopción o intensificación de medidas preventivas para proteger a sus NCC. Además, la información difundida por los medios de comunicación y las redes sociales durante la pandemia^(22)^ también puede haber contribuido a reforzar la percepción familiar sobre la vulnerabilidad de los niños y la gravedad de su cuadro clínico.

En ambos contextos de esta investigación, algunas familias destacaron la perspectiva de normalidad en relación con sus hijos (identidad del niño). Aunque no fue la visión predominante, la normalización fue enfatizada por familias cuyos niños presentaban condiciones crónicas con demandas de cuidado menos complejas. Aun reconociendo las fragilidades impuestas por la condición crónica y la necesidad de cuidados continuos, las familias también expresaron su percepción de normalidad. Este hallazgo sugiere que las familias se encaminan hacia una gestión eficaz de los cuidados, pudiendo ser esta visión un recurso utilizado para cuidar sin limitar al niño a la presencia de una condición crónica.

En relación con la mentalidad de gestión, se identificaron aspectos que facilitan los cuidados comunes a los dos contextos analizados, como la posibilidad de dedicación exclusiva al niño y el acceso al plan de salud. Estas percepciones parecen reflejar la perspectiva de la familia sobre el niño (identidad del niño), reconociendo en él la necesidad de cuidados continuos. La dedicación integral al cuidado surge como estrategia para atender las múltiples demandas, mientras que el acceso al plan de salud asume roles distintos según el período: en el contexto prepandémico, facilitaba la atención domiciliaria, reduciendo los desplazamientos en medio de la sobrecarga familiar; en el contexto de la pandemia, contribuyó al mantenimiento de la atención incluso con la suspensión de los servicios presenciales, lo que demuestra que, aunque no es una facilidad generada por el contexto, su función se ve directamente influenciada por él.

Una revisión sistemática sobre la salud de los padres de niños con condiciones crónicas indica que la dedicación exclusiva al cuidado es un facilitador, debido a la intensa rutina de cuidados en el hogar y consultas especializadas, a menudo incompatible con cualquier actividad remunerada del cuidador principal^(23)^. Sin embargo, esta centralización de las demandas en el cuidador principal puede aumentar su sobrecarga. Este estudio evidenció que los cuidadores eran predominantemente mujeres y que la mayoría de las madres no trabajaban fuera del hogar, dedicándose exclusivamente al hogar y al niño, en consonancia con los hallazgos de la literatura, que señalan que las mujeres dejan de trabajar para quedarse en casa debido a las exigencias impuestas por la condición crónica en la infancia^(24)^.

Se sabe que, durante la pandemia de COVID-19, las medidas de distanciamiento social provocaron una reducción de la red de apoyo de las familias de niños con enfermedades crónicas, lo que hizo que la continuidad del cuidado de estos niños recayera, aún más, en los cuidadores principales^(25)^. Sin embargo, tal como se evidencia en este estudio, la mayor convivencia familiar durante la pandemia favoreció una mayor cohesión familiar, fortaleciendo los vínculos afectivos y la percepción de unión para el cuidado. La mutualidad parental, por lo tanto, contribuye a un manejo más estable y adaptativo, ya que minimiza los conflictos y distribuye responsabilidades, reduciendo la sobrecarga individual y promoviendo una mayor alineación en las acciones^(26)^.

Se observa que el contexto vivido por las familias de niños con condiciones crónicas, especialmente durante la pandemia de COVID-19, impactó directamente en la gestión del cuidado, intensificando las demandas, poniendo de manifiesto las vulnerabilidades y exigiendo la adaptación de las rutinas familiares. Estos hallazgos refuerzan la necesidad de una mirada profesional atenta y especializada, aliada al desarrollo de políticas públicas y estrategias que apoyen efectivamente a las familias en situaciones de emergencia.

Se reconocen algunas limitaciones de este estudio. La recopilación de datos en el contexto de la pandemia se produjo en un momento específico de la pandemia de COVID-19, marcado por una elevada incertidumbre, la ausencia de inmunización y restricciones rigurosas, lo que puede haber influido en las percepciones familiares sobre el niño y el cuidado, limitando la extrapolación de los resultados a otros períodos de la crisis sanitaria. Los estudios que abordan NCC de mayor complejidad, realizados en otros países o en contextos socioculturales distintos, pueden presentar resultados diferentes, en función de las diversas estrategias de enfrentamiento a la pandemia, las especificidades culturales y las políticas de salud adoptadas.

## CONCLUSIÓN

Los hallazgos de este estudio evidencian que la perspectiva familiar sobre el NCC orienta el manejo del cuidado a lo largo del tiempo y es sensible a los cambios contextuales. En el período prepandémico, una perspectiva basada en las potencialidades del niño sustentó prácticas orientadas a la promoción del desarrollo infantil y al mantenimiento de la salud. En el contexto de la pandemia, la COVID-19 se identificó como un agravante adicional a la condición crónica, percibiéndose como una amenaza directa a la vida y contribuyendo a una perspectiva familiar centrada en la fragilidad del niño. Esto dio lugar a un aumento de la vigilancia y a la incorporación de nuevas exigencias de higiene en la rutina familiar. Aun así, el reconocimiento de los aspectos positivos del niño se mantuvo como elemento movilizador del cuidado, favoreciendo el mantenimiento, en el entorno doméstico, de actividades educativas y de rehabilitación por parte de la familia. Estos hallazgos refuerzan el carácter dinámico de la perspectiva familiar y su centralidad en la gestión del cuidado.

A la luz de estos hallazgos, el estudio ofrece implicaciones para la práctica de la enfermería al evidenciar la necesidad de que los enfermeros reconozcan la perspectiva familiar como elemento central en la evaluación y la planificación del cuidado de los niños con NCC, especialmente ante cambios en sus contextos de vida, como las crisis sanitarias.

El uso de marcos teóricos, como el FMSF, puede ayudar a los profesionales a identificar cómo los cuidadores perciben al niño y organizan la atención en la vida cotidiana, favoreciendo las intervenciones centradas en la familia. En el ámbito de las políticas públicas, los resultados refuerzan la importancia de mantener la continuidad de la atención, fortalecer las redes de apoyo y organizar respuestas asistenciales que consideren las demandas específicas de las familias en situaciones de crisis, especialmente cuando estas producen cambios en la oferta de los servicios que necesitan los niños. Se necesitan investigaciones futuras para profundizar en la comprensión de la perspectiva familiar en otros contextos y a lo largo del tiempo.

## DISPONIBILIDAD DE DATOS

El conjunto de datos que respaldan las conclusiones de este estudio está disponible previa solicitud al autor correspondiente.
